# Supplementary material for: SemanticST: A Scalable Multi‐Contextual Graph Learning Framework for Uncovering Spatial Niches and Robust Multi‐Sample Integration in Spatial Transcriptomics
Source: Adv Sci (Weinh). 2026 Aug 31:e77003. Online ahead of print. doi: 10.1002/advs.77003 (PMC13528690; doi:10.1002/advs.77003)
Supplement: Supplementary file 1 [file ADVS-9999-e77003-s001.pdf]

SemanticST: A Scalable Multi-Contextual Graph Learning Framework for Uncovering Spatial Niches and Robust Multi-Sample Integration in Spatial Transcriptomics

Roxana Zahedi*[1], Ahmadreza Argha[2,3], Nona Farbehi[2,3], Ivan Bakhshayeshi[1], Thantrira Porntaveetus†[4,5], Youqiong Ye[6], Nigel H. Lovell[2,3], and Hamid Alinejad-Rokny‡[1,7]

[1]UNSW BioMedical Machine Learning Lab (BML), School of Biomedical Engineering, UNSW Sydney, Sydney, 2052, NSW, Australia
[2]School of Biomedical Engineering, UNSW Sydney, Sydney, 2052, NSW, Australia
[3]Tyree Institute of Health Engineering (IHealthE), UNSW Sydney, Sydney, 2052, NSW, Australia
[4]Center of Excellence in Precision Medicine and Digital Health, Department of Physiology, Faculty of Dentistry, Chulalongkorn University, Bangkok, 10330, Thailand
[5]Clinic of General, Special Care and Geriatric Dentistry, Center for Dental Medicine, University of Zurich, Zurich, Switzerland
[6]Center for Immune-Related Diseases, Shanghai Institute of Immunology, Shanghai Jiao Tong University School of Medicine, Shanghai, 200001, China
[7]Visiting Scholar (Collaborative Projects), Center of Excellence in Precision Medicine and Digital Health, Chulalongkorn University, Bangkok, Thailand

*r.zahedi@unsw.edu.au
†Corresponding author
‡h.alinejad@unsw.edu.au; Corresponding author

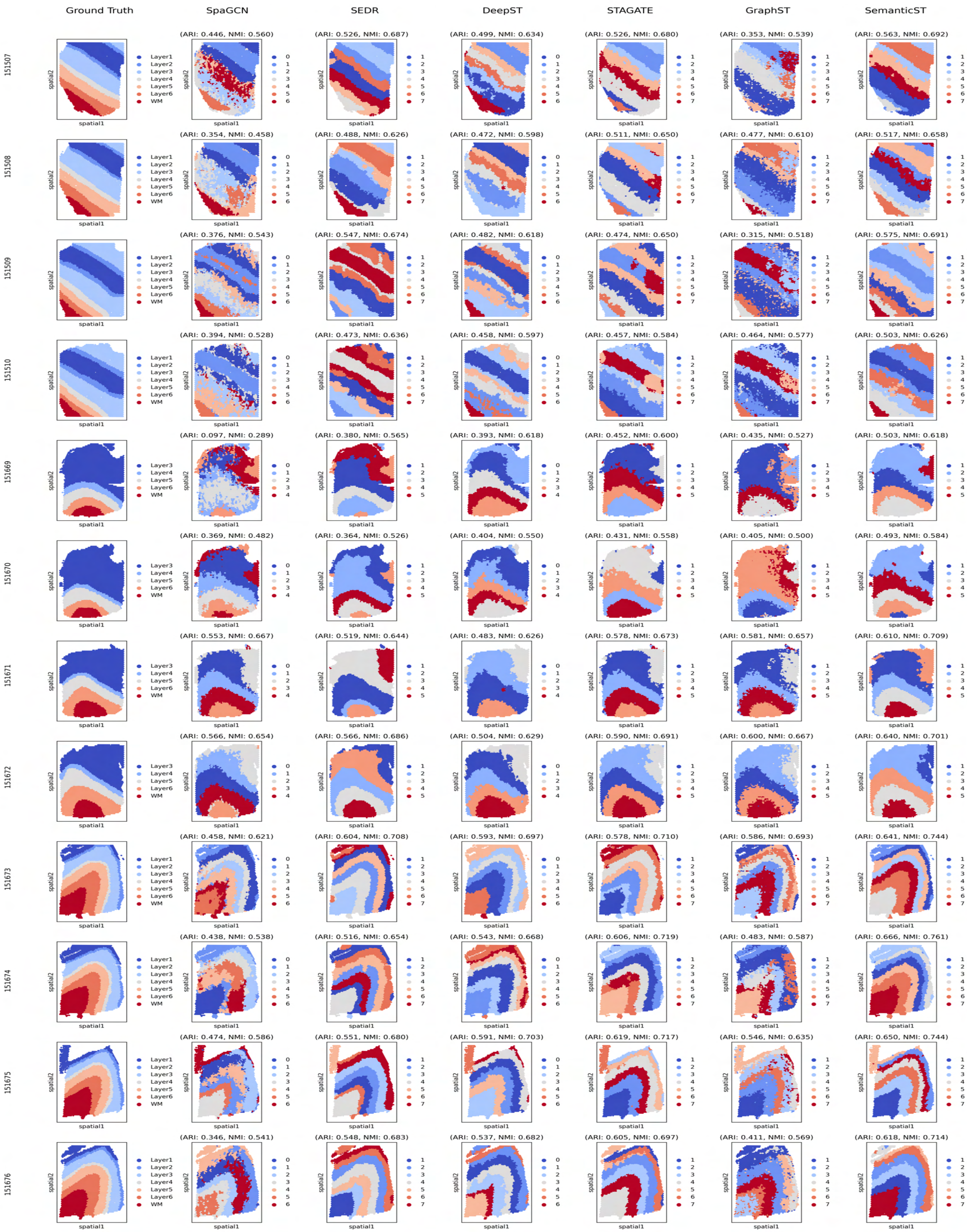

Ground Truth
SpaGCN
SEDR
DeepST
STAGATE
GraphST
SemanticST
151507
(ARI: 0.446, NMI: 0.560)
(ARI: 0.526, NMI: 0.687)
(ARI: 0.499, NMI: 0.634)
(ARI: 0.526, NMI: 0.680)
(ARI: 0.353, NMI: 0.539)
(ARI: 0.563, NMI: 0.692)
151508
(ARI: 0.354, NMI: 0.458)
(ARI: 0.488, NMI: 0.626)
(ARI: 0.472, NMI: 0.598)
(ARI: 0.511, NMI: 0.650)
(ARI: 0.477, NMI: 0.610)
(ARI: 0.517, NMI: 0.658)
151509
(ARI: 0.376, NMI: 0.543)
(ARI: 0.547, NMI: 0.674)
(ARI: 0.482, NMI: 0.618)
(ARI: 0.474, NMI: 0.650)
(ARI: 0.315, NMI: 0.518)
(ARI: 0.575, NMI: 0.691)
151510
(ARI: 0.394, NMI: 0.528)
(ARI: 0.473, NMI: 0.636)
(ARI: 0.458, NMI: 0.597)
(ARI: 0.457, NMI: 0.584)
(ARI: 0.464, NMI: 0.577)
(ARI: 0.503, NMI: 0.626)
151669
(ARI: 0.097, NMI: 0.289)
(ARI: 0.380, NMI: 0.565)
(ARI: 0.393, NMI: 0.618)
(ARI: 0.452, NMI: 0.600)
(ARI: 0.435, NMI: 0.527)
(ARI: 0.503, NMI: 0.618)
151670
(ARI: 0.369, NMI: 0.482)
(ARI: 0.364, NMI: 0.526)
(ARI: 0.404, NMI: 0.550)
(ARI: 0.431, NMI: 0.558)
(ARI: 0.405, NMI: 0.500)
(ARI: 0.493, NMI: 0.584)
151671
(ARI: 0.553, NMI: 0.667)
(ARI: 0.519, NMI: 0.644)
(ARI: 0.483, NMI: 0.626)
(ARI: 0.578, NMI: 0.673)
(ARI: 0.581, NMI: 0.657)
(ARI: 0.610, NMI: 0.709)
151672
(ARI: 0.566, NMI: 0.654)
(ARI: 0.566, NMI: 0.686)
(ARI: 0.504, NMI: 0.629)
(ARI: 0.590, NMI: 0.691)
(ARI: 0.600, NMI: 0.667)
(ARI: 0.640, NMI: 0.701)
151673
(ARI: 0.458, NMI: 0.621)
(ARI: 0.604, NMI: 0.708)
(ARI: 0.593, NMI: 0.697)
(ARI: 0.578, NMI: 0.710)
(ARI: 0.586, NMI: 0.693)
(ARI: 0.641, NMI: 0.744)
151674
(ARI: 0.438, NMI: 0.538)
(ARI: 0.516, NMI: 0.654)
(ARI: 0.543, NMI: 0.668)
(ARI: 0.606, NMI: 0.719)
(ARI: 0.483, NMI: 0.587)
(ARI: 0.666, NMI: 0.761)
151675
(ARI: 0.474, NMI: 0.586)
(ARI: 0.551, NMI: 0.680)
(ARI: 0.591, NMI: 0.703)
(ARI: 0.619, NMI: 0.717)
(ARI: 0.546, NMI: 0.635)
(ARI: 0.650, NMI: 0.744)
151676
(ARI: 0.346, NMI: 0.541)
(ARI: 0.548, NMI: 0.683)
(ARI: 0.537, NMI: 0.682)
(ARI: 0.605, NMI: 0.697)
(ARI: 0.411, NMI: 0.569)
(ARI: 0.618, NMI: 0.714)
Layer1
Layer2
Layer3
Layer4
Layer5
Layer6
WM
spatial1
spatial2

Figure S1: Identification of spatial domains using SemanticST, with a visual comparison to Ground Truth, SpaGCN, SEDR, DeepST, STAGATE, and GraphST across 12 sections of the DLPFC dataset.

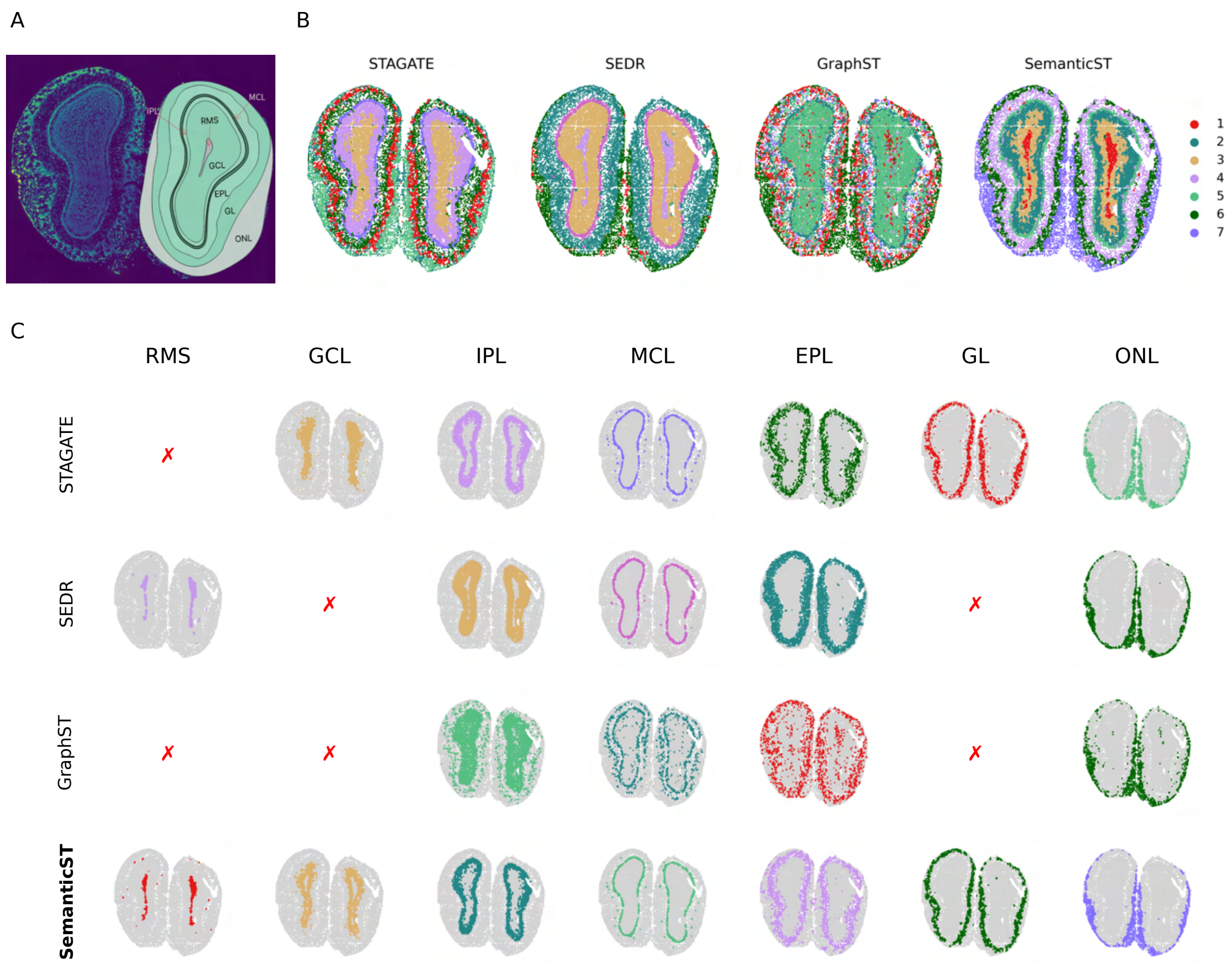

Figure S2: A Tissue annotation derived from the study by Fu et al., showing corresponding laminar layers including RMS, GCL, IPL, MCL, EPL, and ONL. **B** Clustering results from STAGATE, SEDR, GraphST, and SemanticST on the mouse olfactory bulb dataset. **C** Detailed visual comparison of spatial domains identified by SemanticST and other methods.

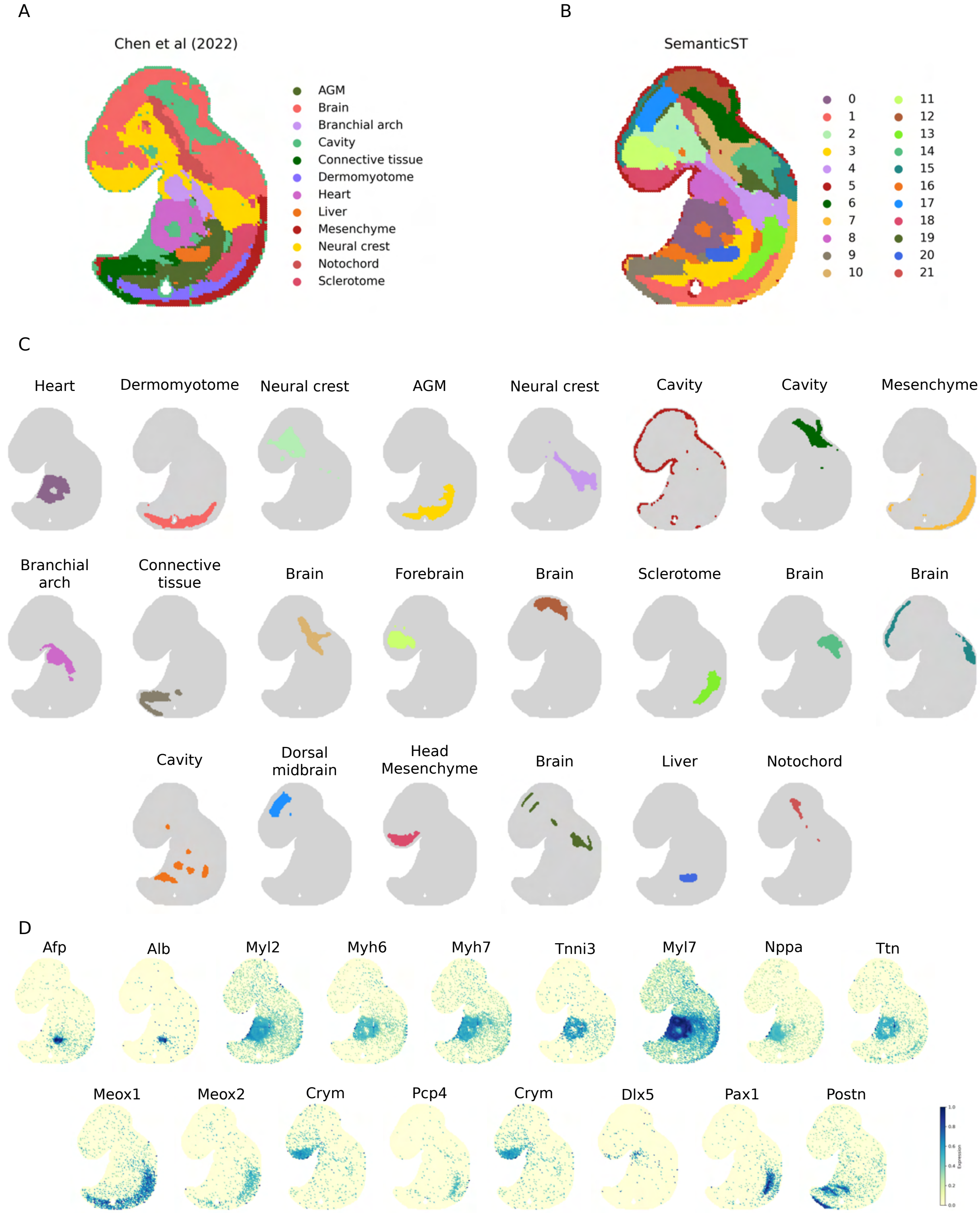

Figure S3: Clustering analysis of spatial data from the E9.5 mouse embryo Stereo-seq dataset. A. Annotations based on the original study. **B.** Spatial clustering results from SemanticST. **C.** Separate visualization of each of the 22 spatial domains identified by SemanticST. **D.** Visualization of marker genes defining each spatial domain.

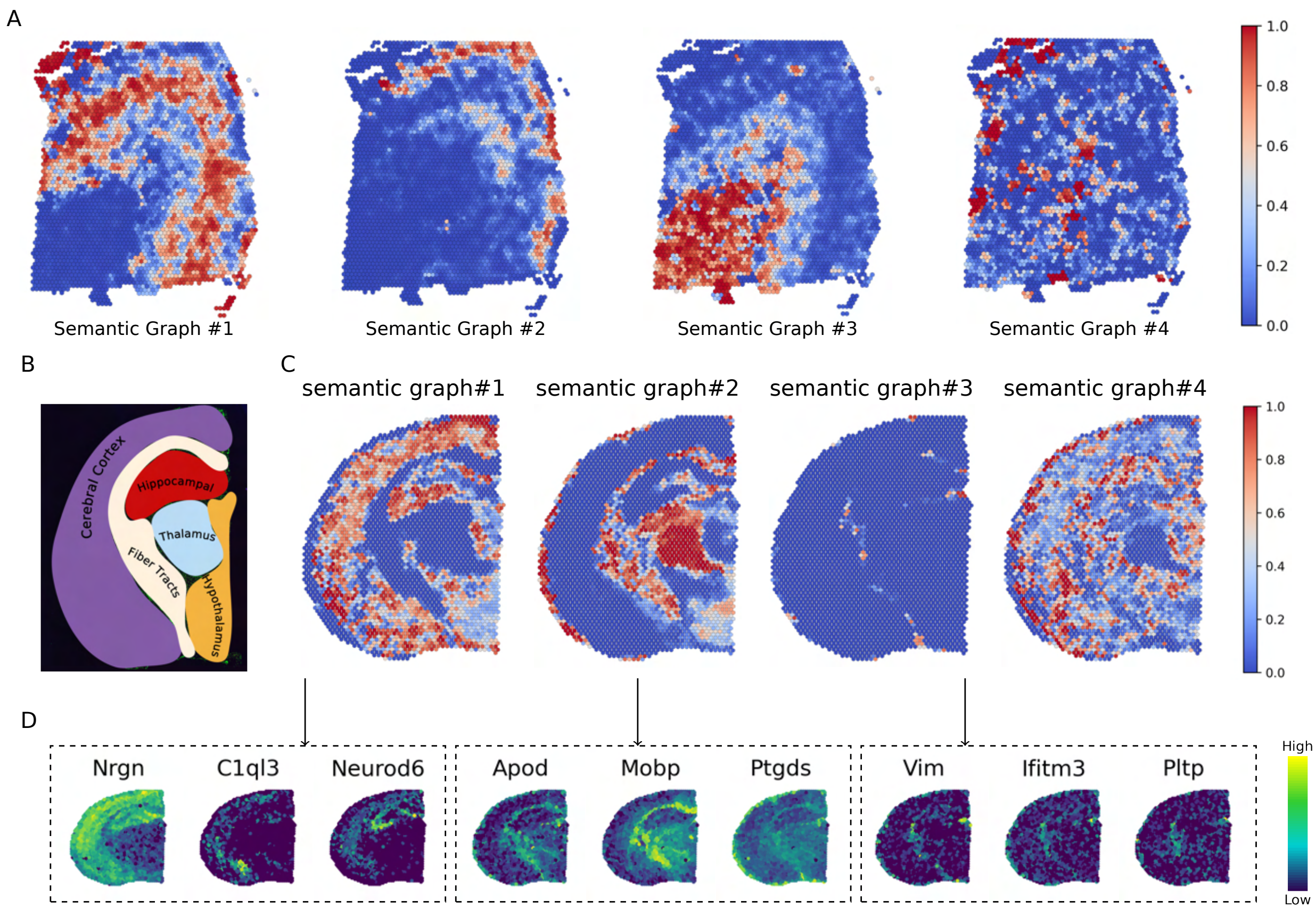

Figure S4: A. Heatmap of attention scores from four semantic graphs generated by SemanticST from DLPFC dataset.**B** Mouse brain Visium data annotated into five main regions based on the Allen Reference Atlas. **C** Heatmap illustrating learned attention scores from four semantic graphs in the mouse brain Visium dataset. **D** Top three differentially expressed genes in semantic graphs, determined by Wilcoxon rank-sum test (false discovery rate (FDR)$\geq$ 0.1 and log_2 fold change ≥ 1).

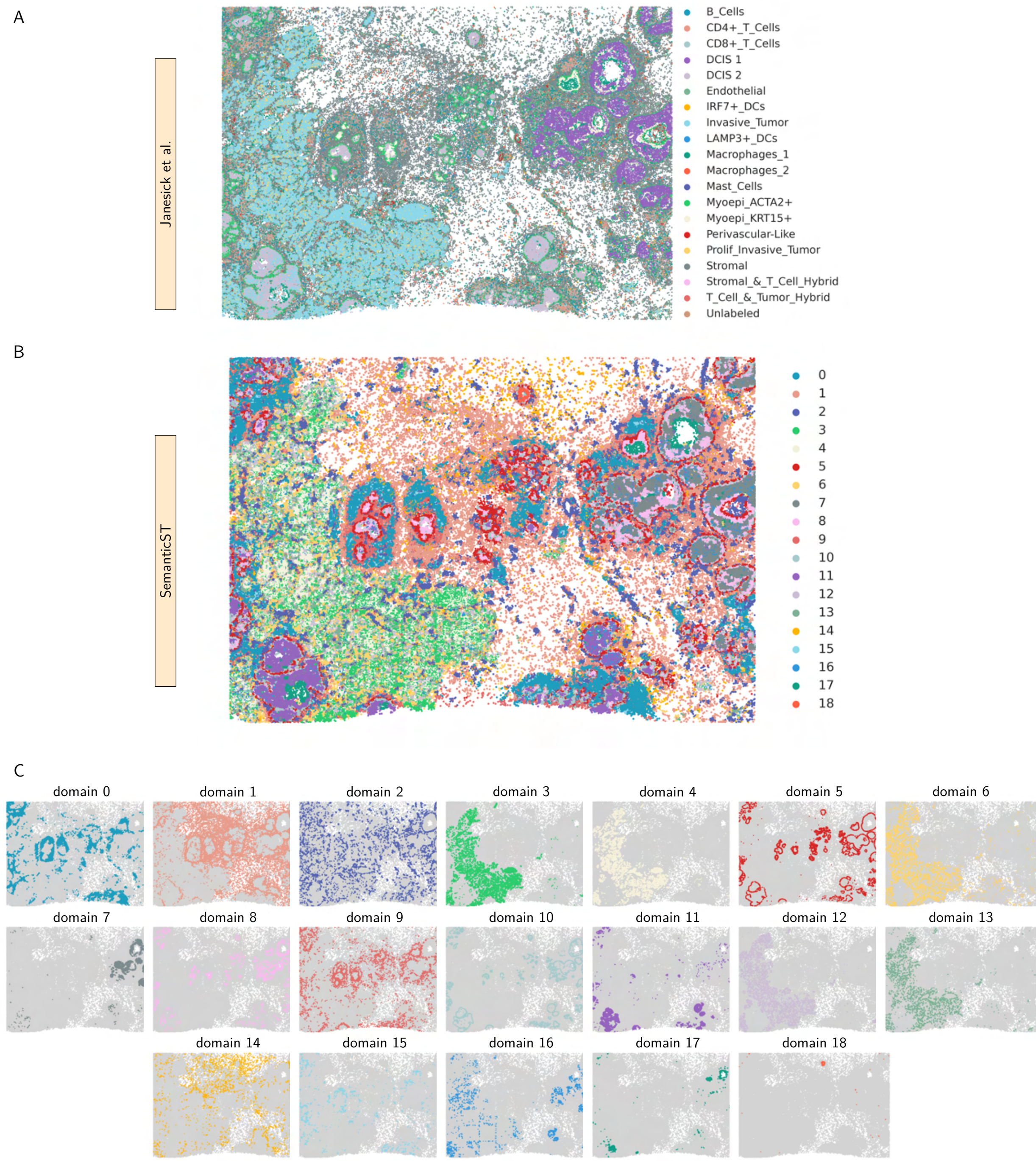

Figure S5: Clustering results on Xenium data from breast cancer. **A.** Clustering results of original annotation. **B.** Clustering results of SemanticST. **C.** Separate visualization of the 19 clusters identified by SemanticST.

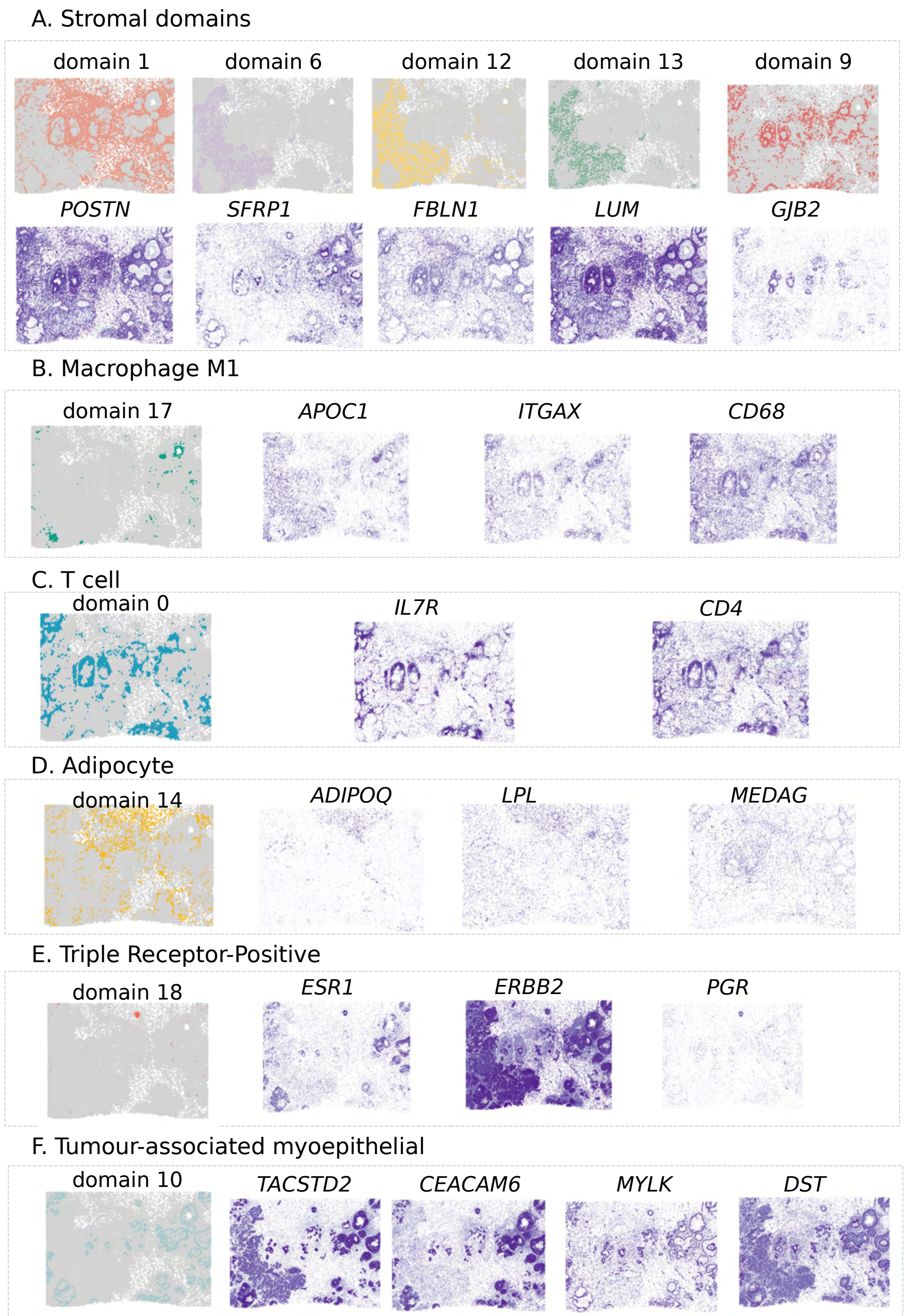

Figure S6: Identification of A. Stromal, B. Macrophage, C. T cell, D. Adipocyte domains, E. Triple receptor positive, and F. Tumour-associated myoepithelial by SemanticST, along with their associated highly expressed genes.

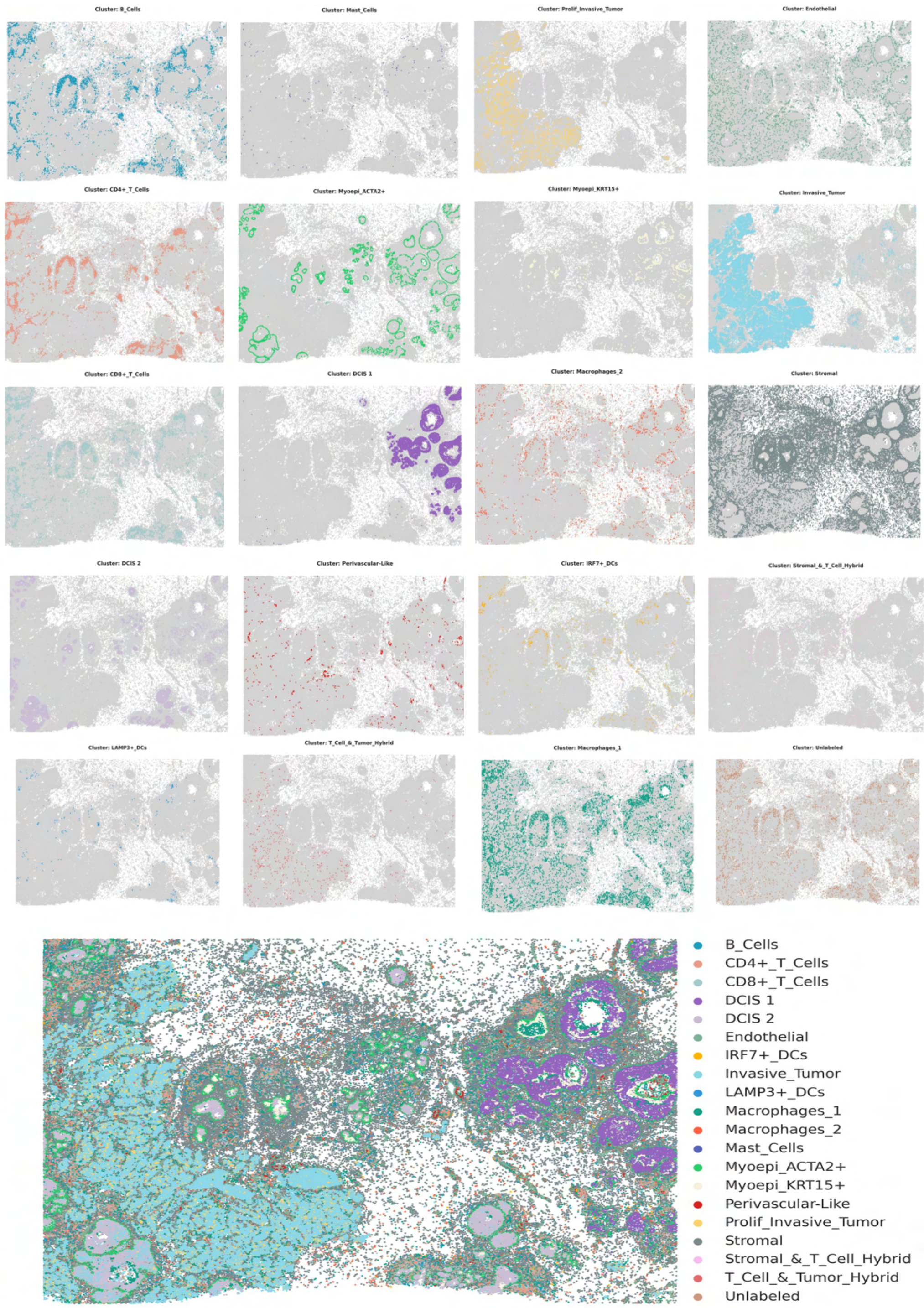

Figure S7: Separate visualization of the 20 clusters identified by Janesick et al.

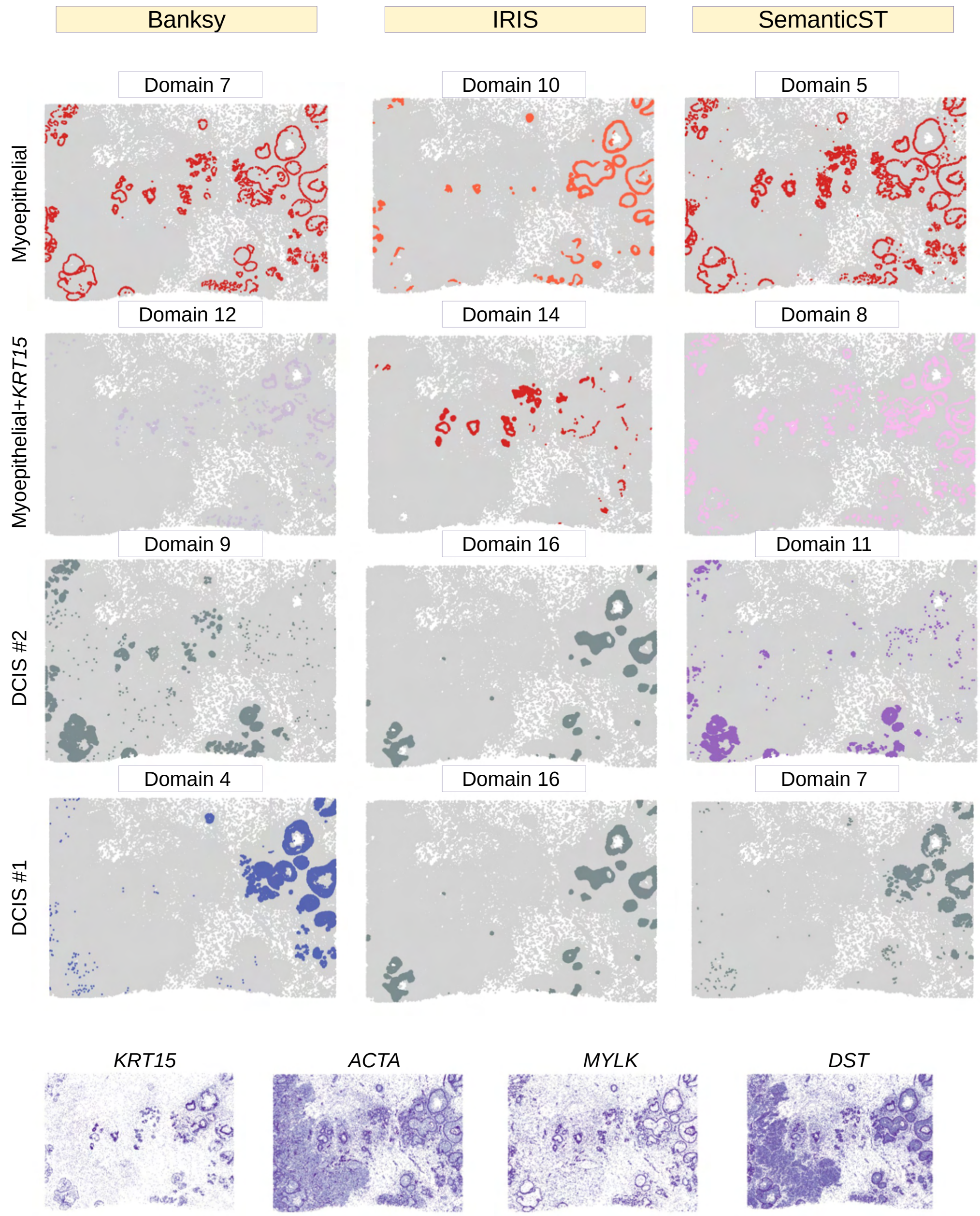

Figure S8: Myoepithelial layer and DCIS regions identified by Banksy, IRIS, and SemanticST

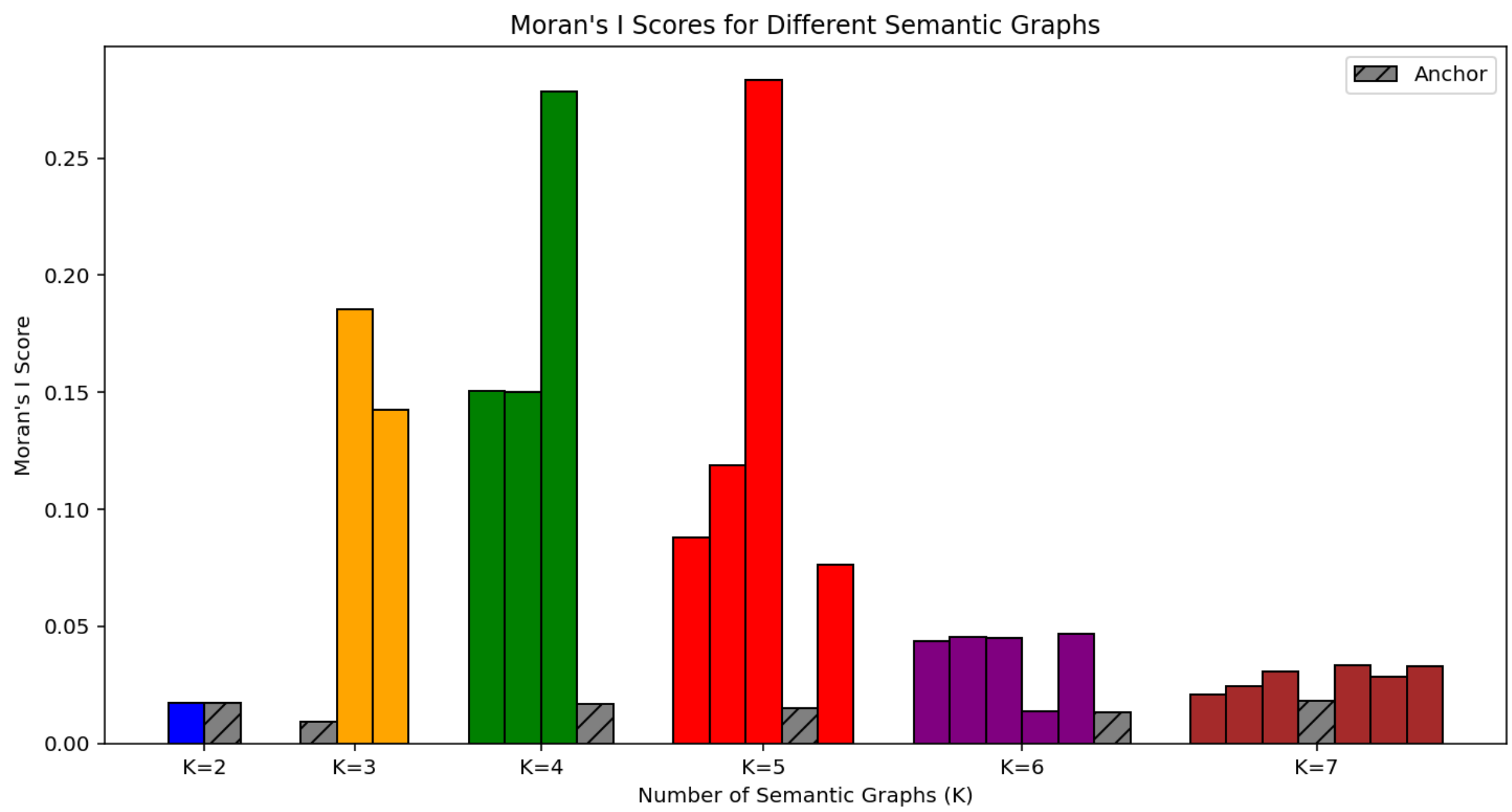

Figure S9: Moran's I scores for different number of semantic graphs (k=2,...,7).

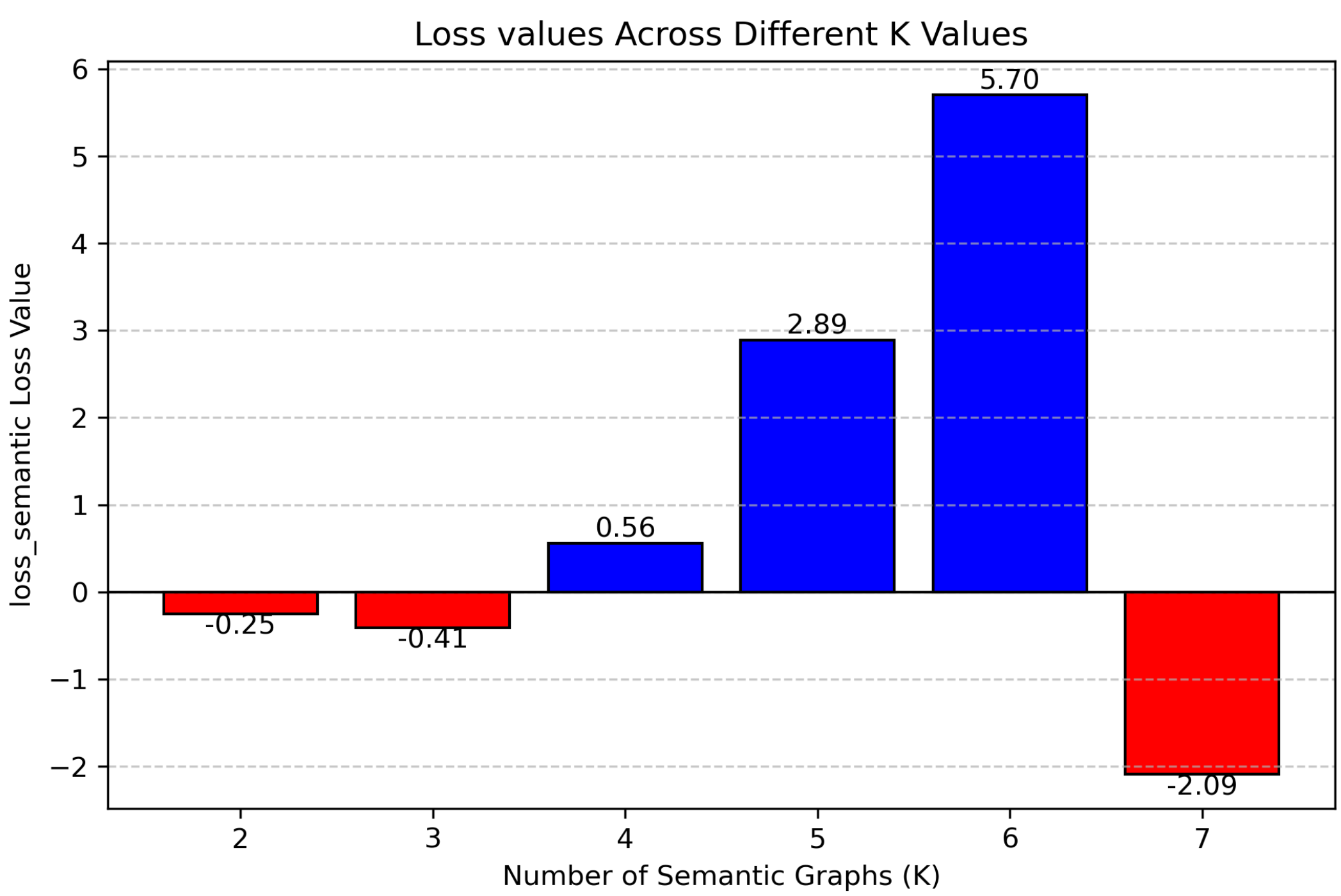

Figure S10: Loss values for varying numbers of semantic graphs

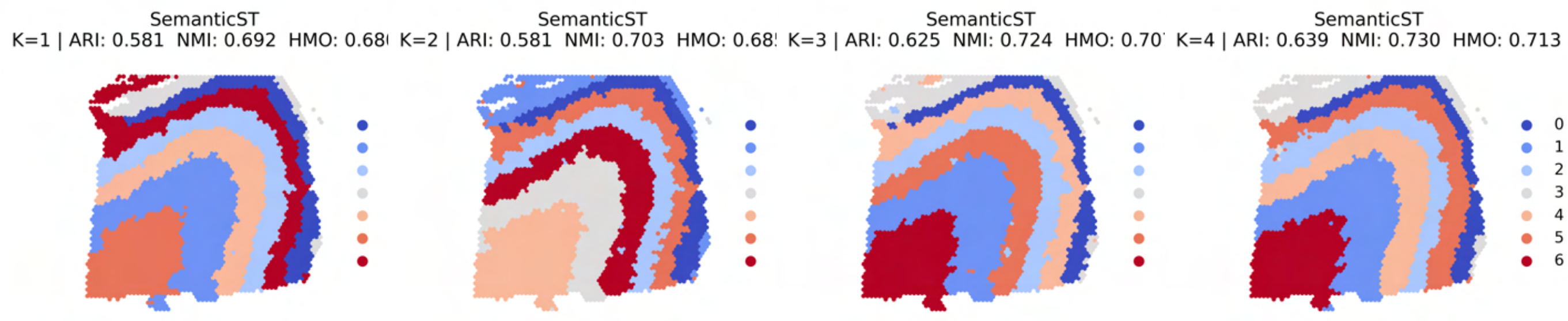

(a) one weighted graph **(b)** $K = 2$ **(c)** $K = 3$ **(d)** $K = 4$

Figure S11: Effect of the number of semantic graphs (K) on spatial domain separation in the DLPFC dataset. When a single weighted graph is used, domains 5 and 7 exhibit noticeable mixing. Increasing K leads to improved spatial separation and clearer domain boundaries.

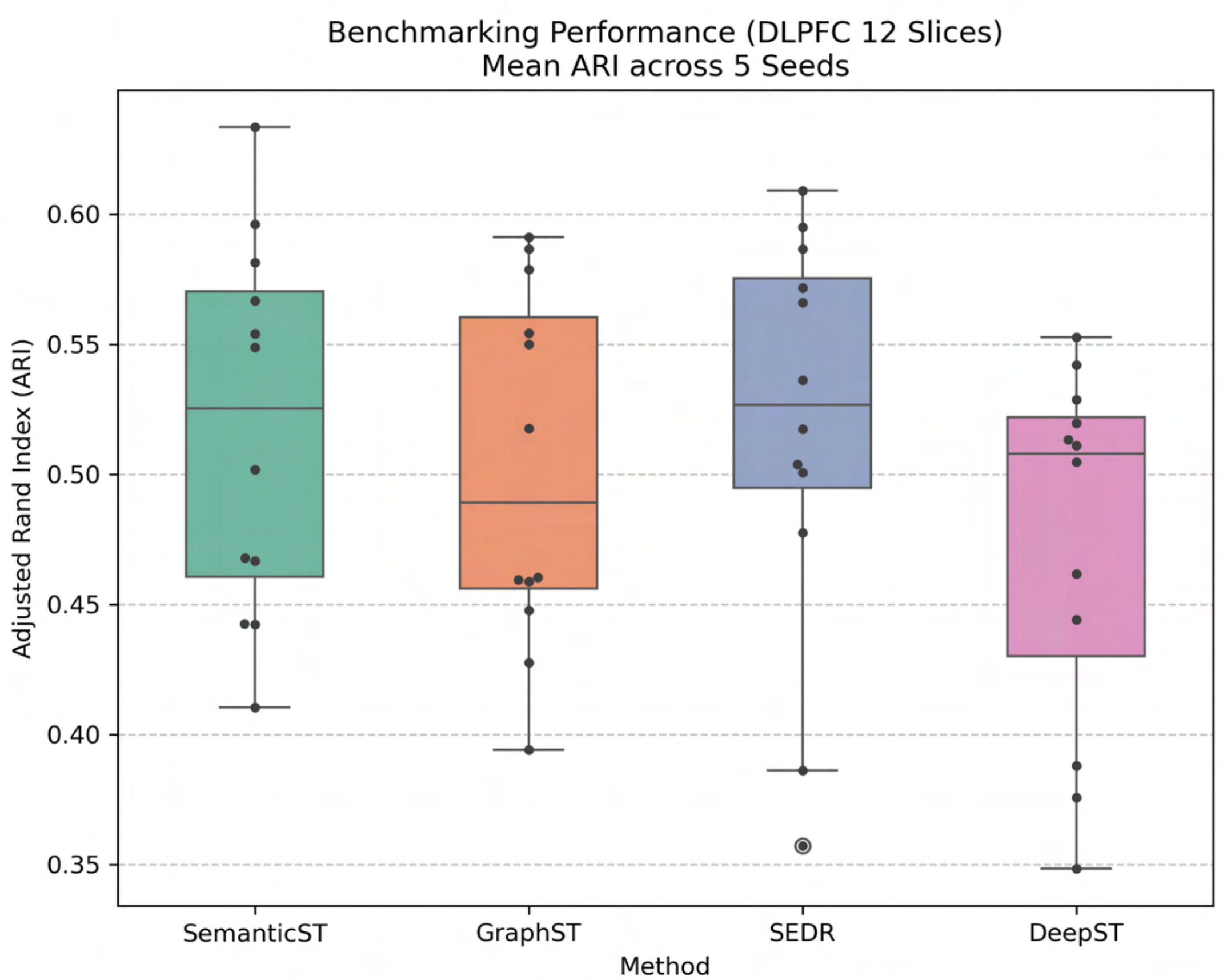

Figure S12: Comparison of clustering performance measured by ARI across all slices with different random seeds.

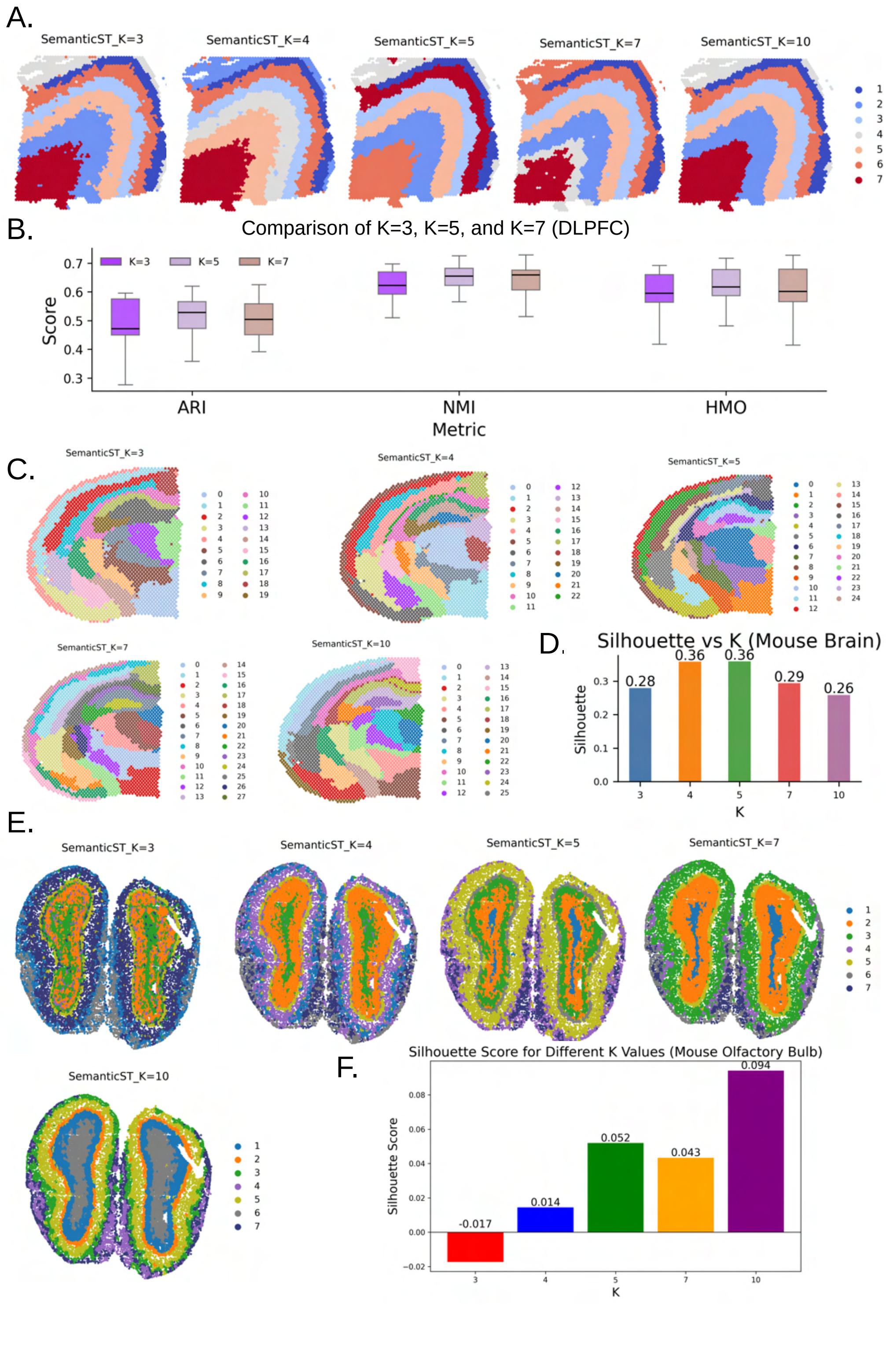

Figure S13: (A) Spatial domain plot of DLPFC slice #151673 under different values of k used in the KNN graph. (B) Comparison of clustering performance metrics (ARI, NMI, and HMO) across 12 DLPFC sections for different k values. (C) Visual comparison of spatial domains in the mouse brain Visium dataset under different k values, and (D) the corresponding silhouette scores. (E) Visual comparison of spatial domains in the mouse olfactory bulb Stereo-seq dataset under different k values, and (F) the corresponding silhouette scores.

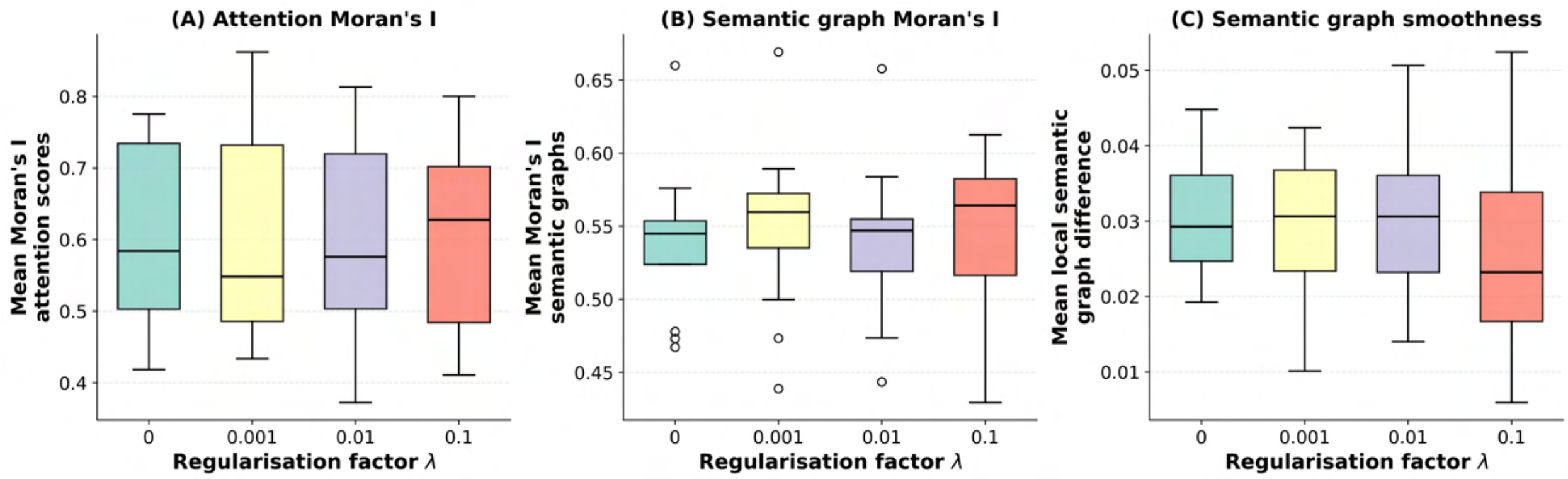

Figure S14: Sensitivity analysis of the regularisation factor λ across spatial semantic metrics. Evaluation was performed across 12 sections of the DLPFC dataset. (A) Moran's I of the encoder-learned attention scores remains broadly stable across the tested λ values, indicating that the final attention scores are not highly sensitive to this parameter. (B) Moran's I of the learned semantic graphs shows stronger spatial autocorrelation at higher regularisation, particularly for $\lambda = 0.1$. (C) Local semantic graph difference measures smoothness between neighbouring node-level semantic scores, where lower values indicate smoother semantic graphs. Overall, $\lambda = 0.1$ provides stronger semantic graph spatial coherence while maintaining stable attention-level spatial structure.

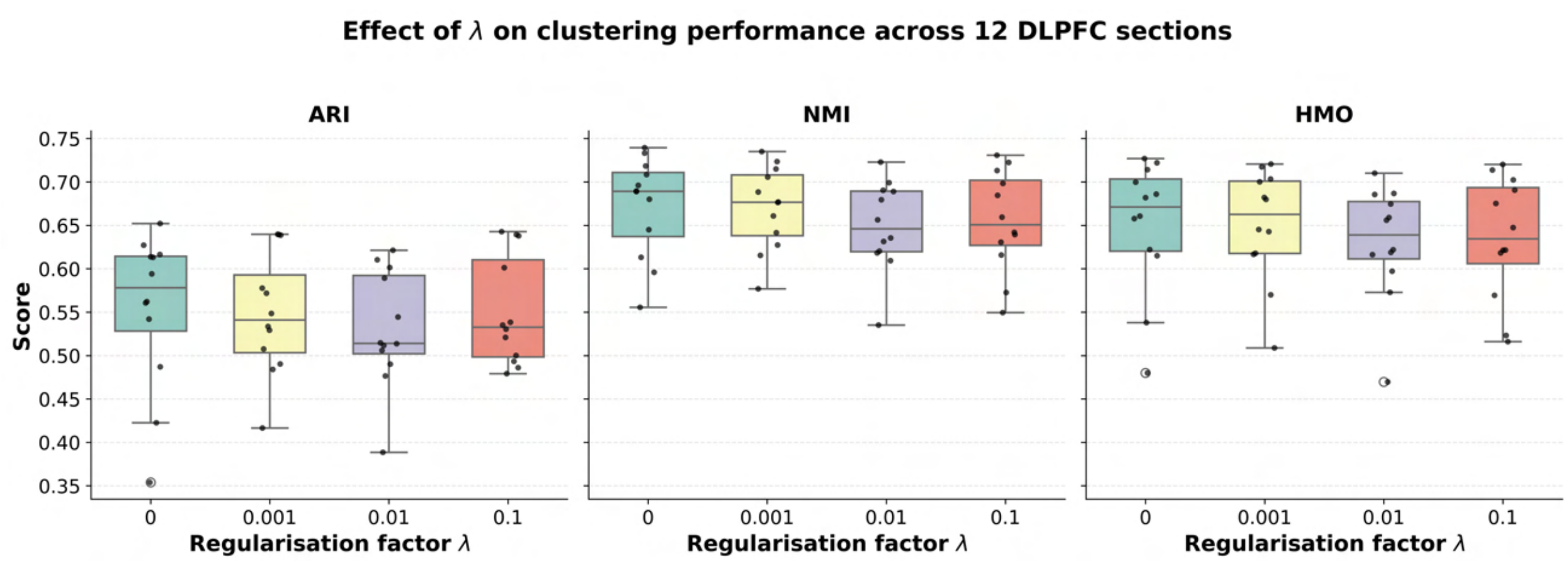

Figure S15: Impact of λ on biological domain characterization across 12 DLPFC sections. The boxplots represent the distribution of (Left) Adjusted Rand Index (ARI), (Center) Normalized Mutual Information (NMI), and (Right) Homogeneity (HMO) scores.

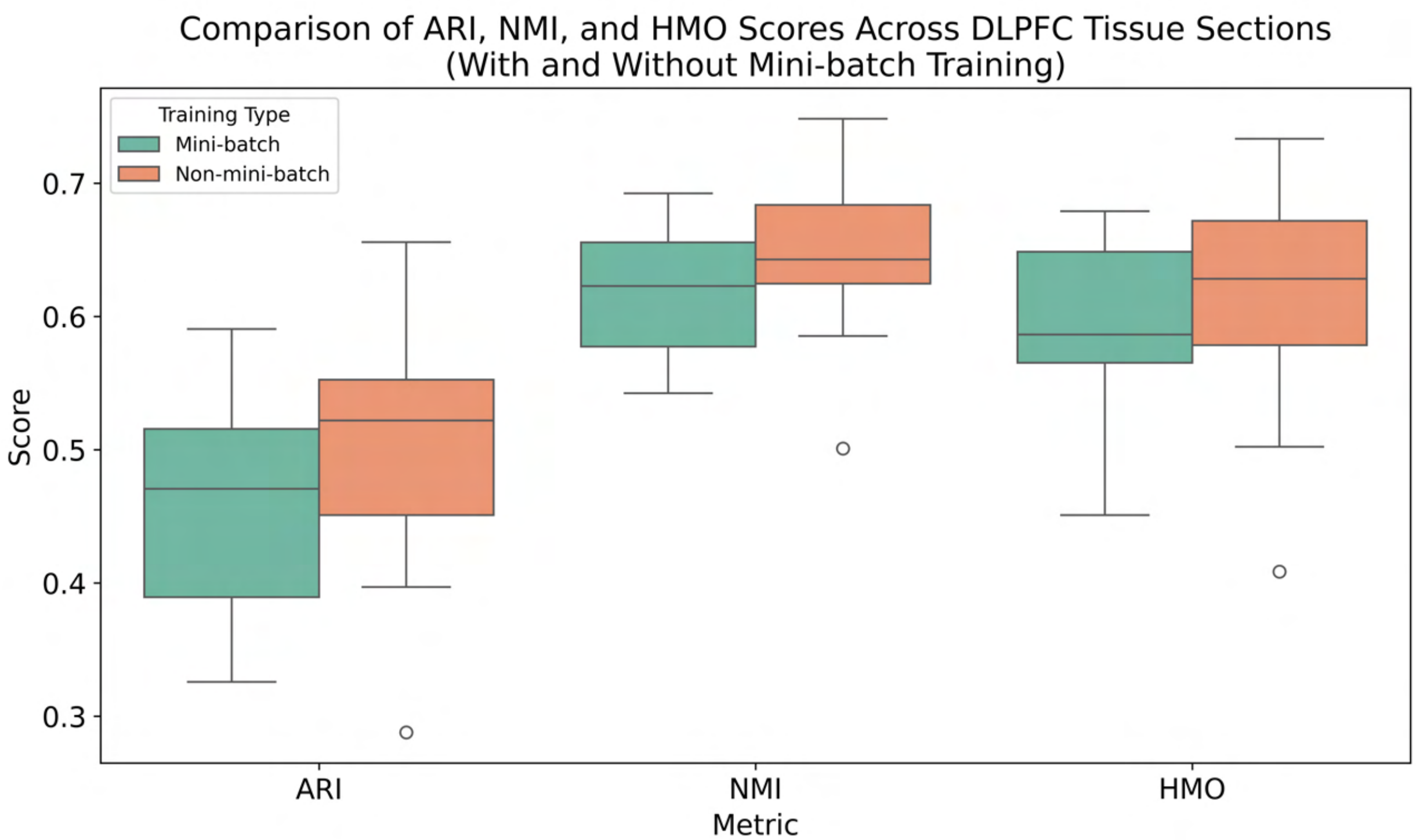

Figure S16: Comparison of ARI, NMI, and HMO Scores Across DLPFC Tissue Sections (With and Without Mini-batch Training)

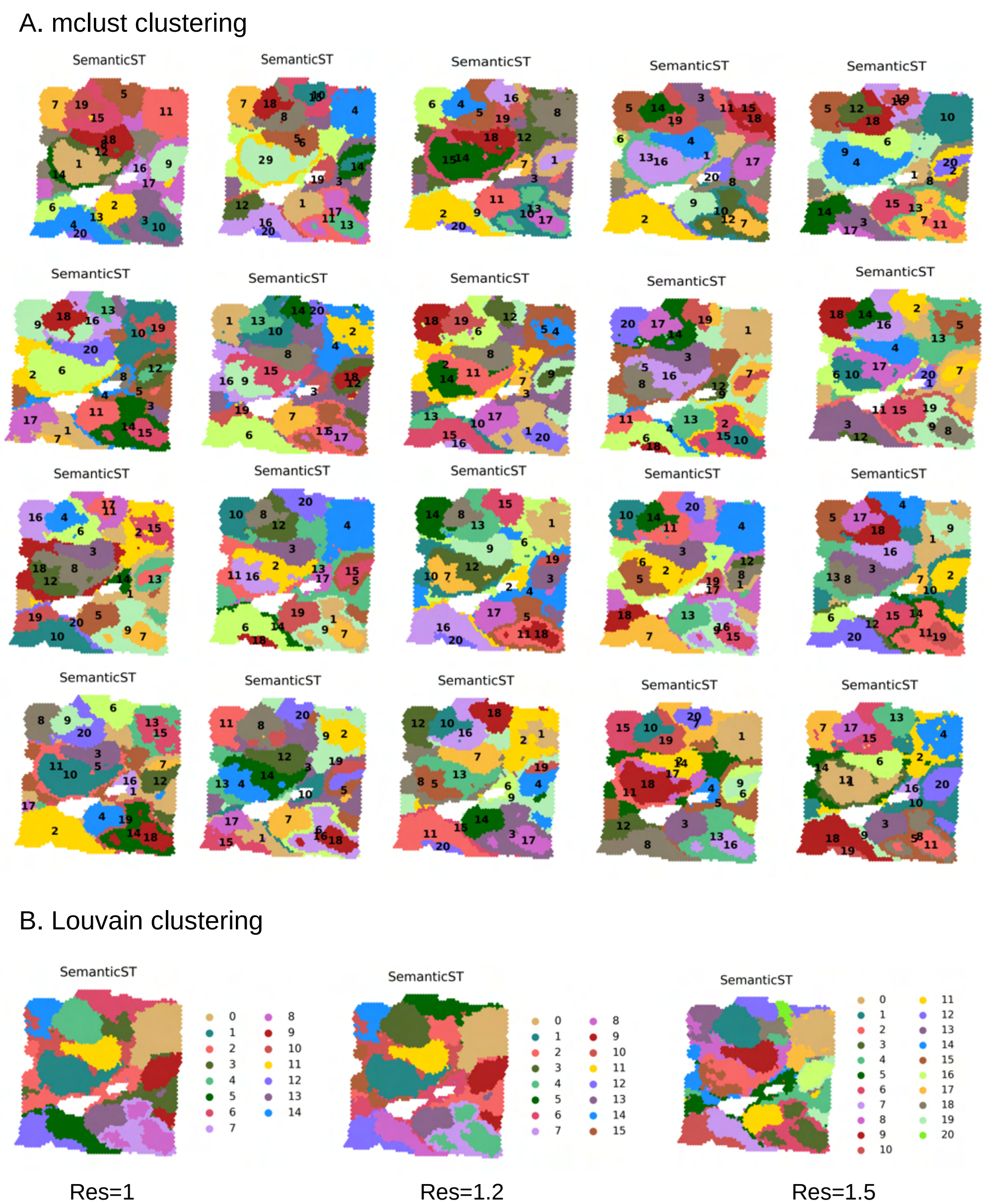

Figure S17: Stability analysis of the Visium breast cancer dataset using (A) Mclust clustering with $k = 20$ across 20 different random seeds and (B) Louvain clustering across multiple resolution values.

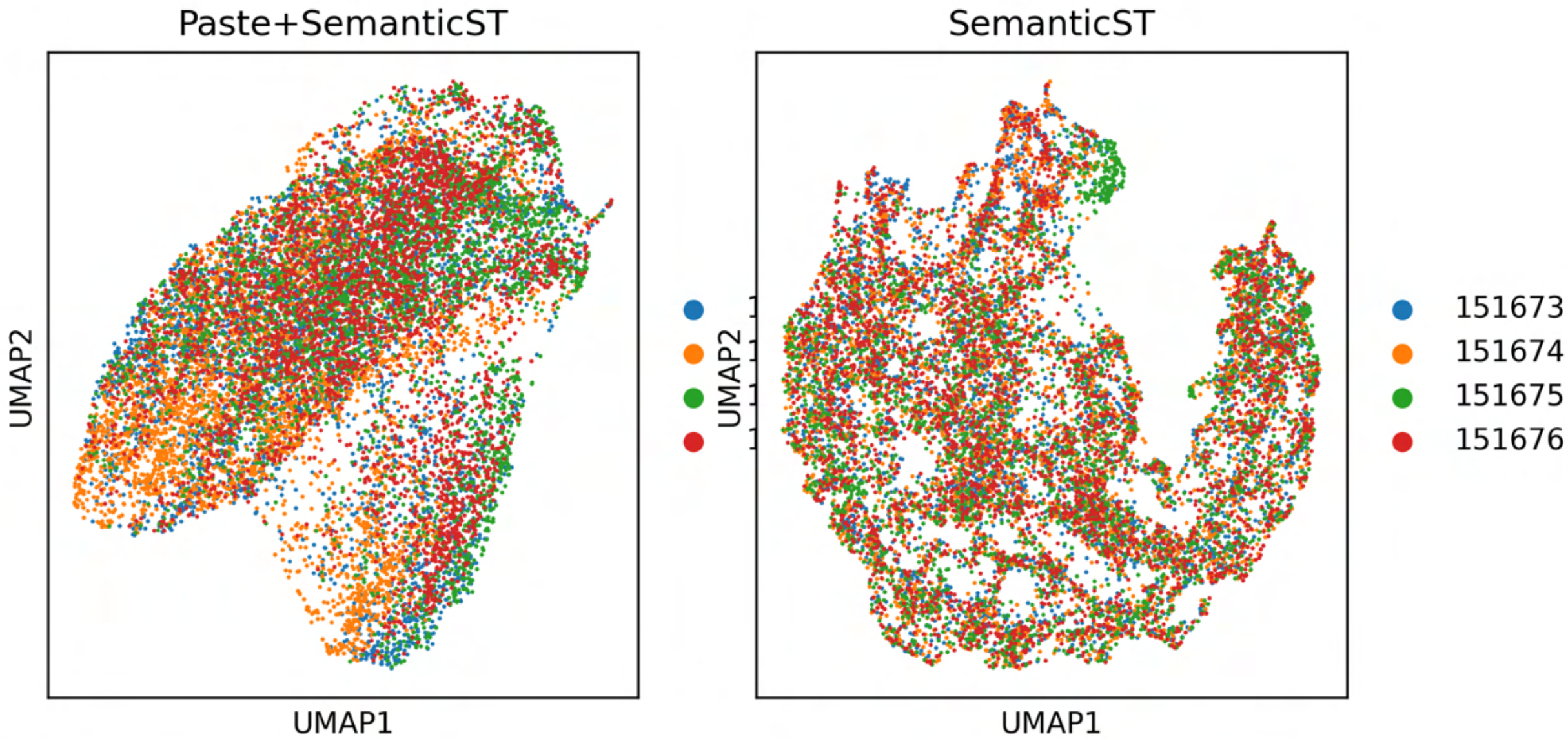

Figure S18: UMAPs displaying the integration results Paste+SemanticST and block-diagonal SemanticST across four sections of the human dorsolateral prefrontal cortex (DLPFC) dataset, specifically sections #151673, #151674, #151675, and #151676.

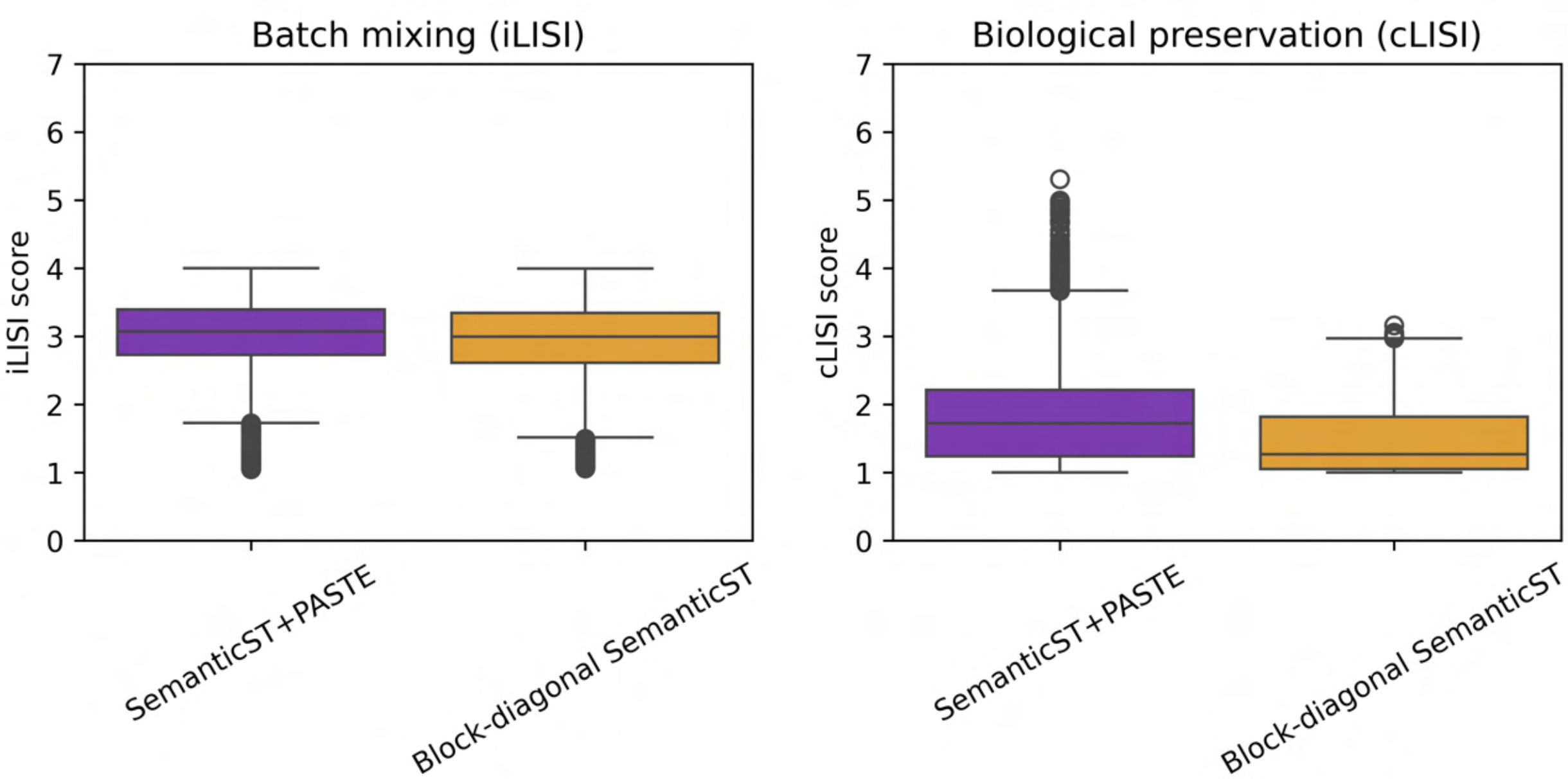

Figure S19: iLISI and cLISI scores for the integration results of the four DLPFC sections using the latent representations generated by Paste+SemanticST and block-diagonal SemanticST.

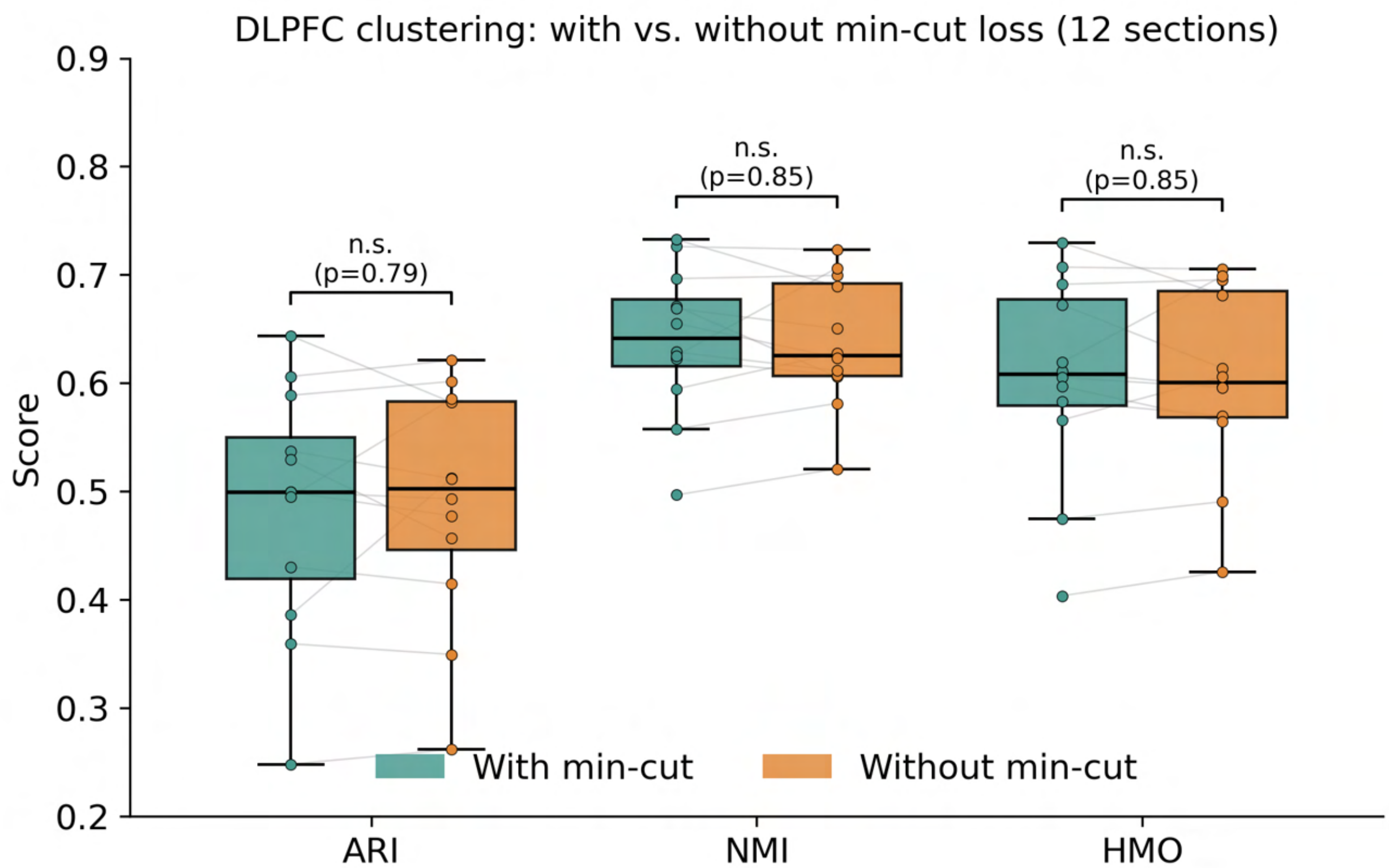

Figure S20: Clustering performance across the 12 DLPFC sections with and without the min-cut loss. Paired Wilcoxon signed-rank test across the 12 sections: ARI $p = 0.79$, NMI $p = 0.85$, HMO $p = 0.85$ (n.s.).

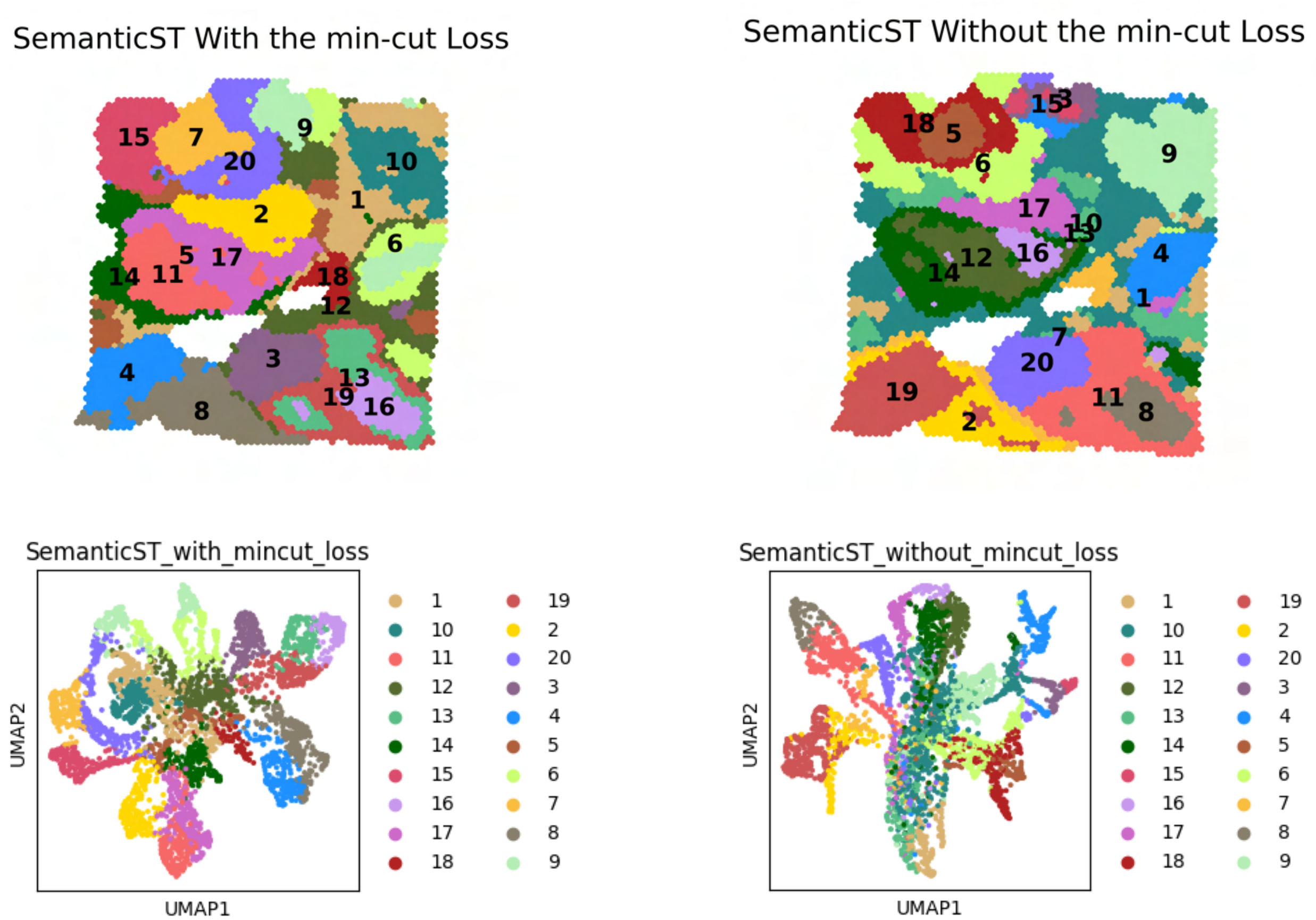

Figure S21: Spatial plot of human breast cancer Visium data (top) and UMAP plot of the SemanticST embedding space (bottom), comparing results with and without the min-cut loss function.

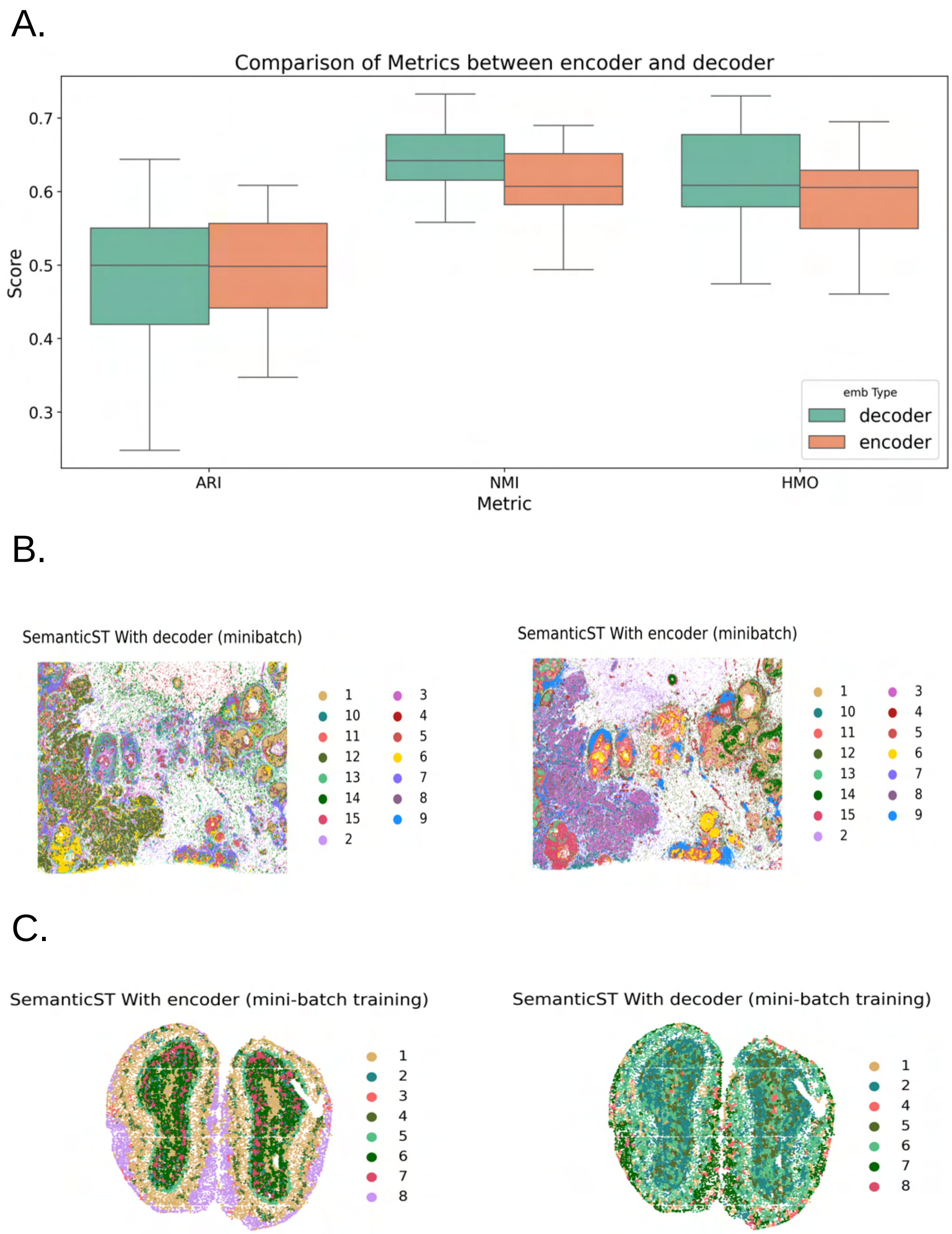

Figure S22: A. Comparison of metrics (ARI, NMI, HMO) between encoder and decoder outputs on the DLPFC dataset across 12 sections. B. Spatial clustering results with decoder (left) and encoder (right) outputs on the Xenium human breast cancer dataset. C. Spatial clustering with encoder (left) and decoder (right) outputs in mini-batch training on the mouse olfactory bulb dataset.

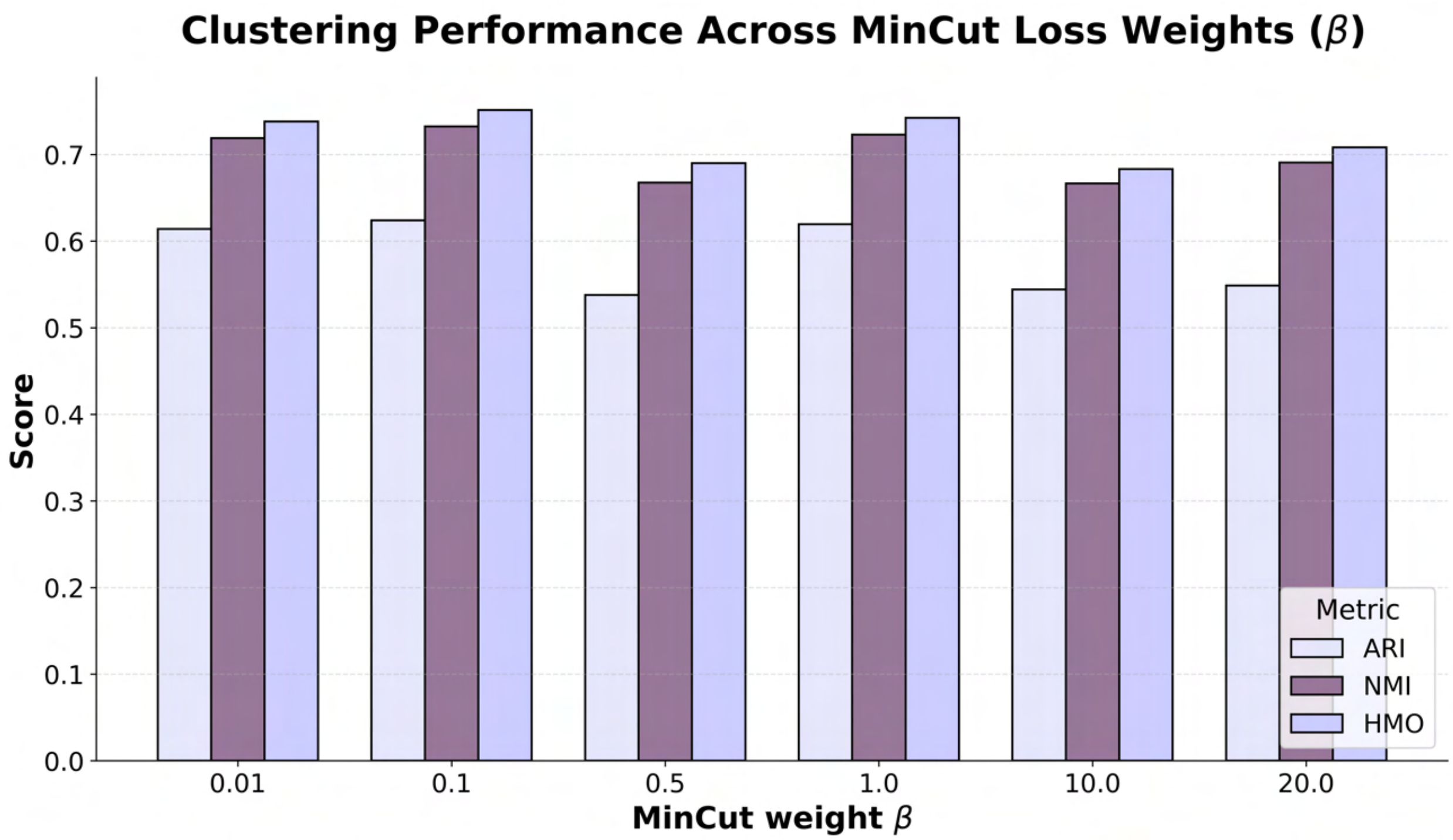

Figure S23: Sensitivity analysis of the min-cut loss weight on DLPFC section 151673. The reconstruction loss weight was fixed at $\beta_1 = 10$, while the min-cut loss weight β_2 was varied across different values. Clustering performance remained relatively stable for small-to-moderate values of β_2, with a gradual decrease at larger values.

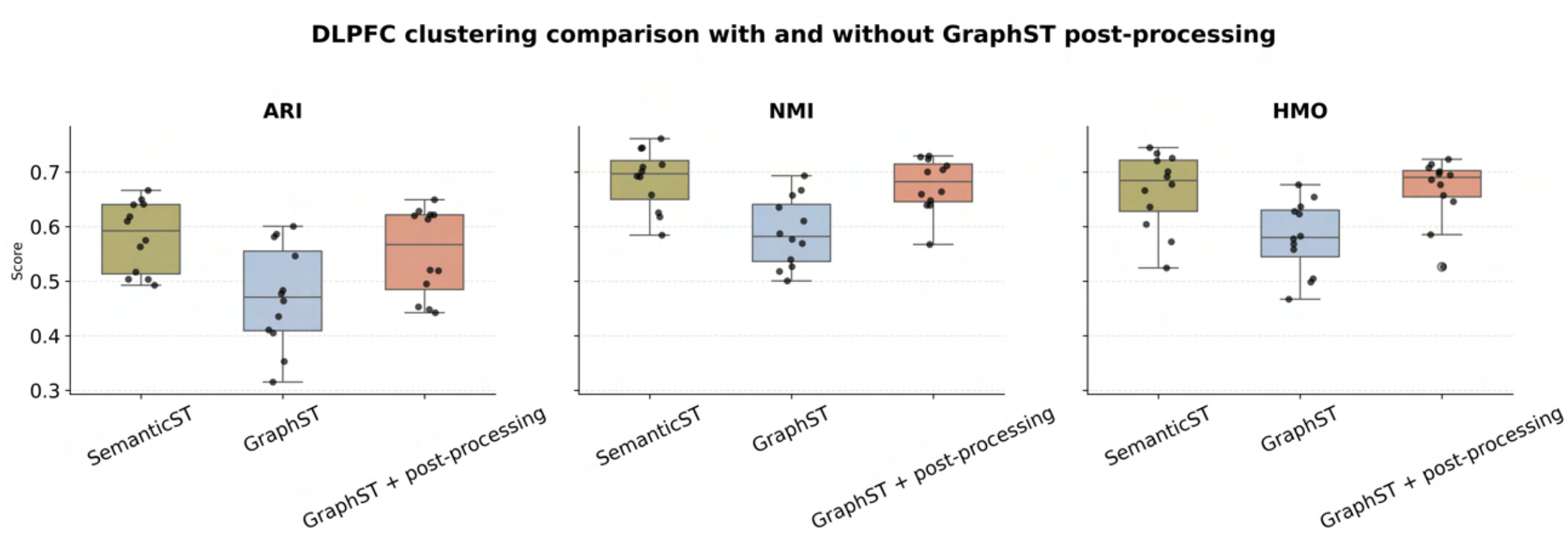

Figure S24: Comparison of SemanticST with GraphST before and after post-processing across the 12 DLPFC sections.

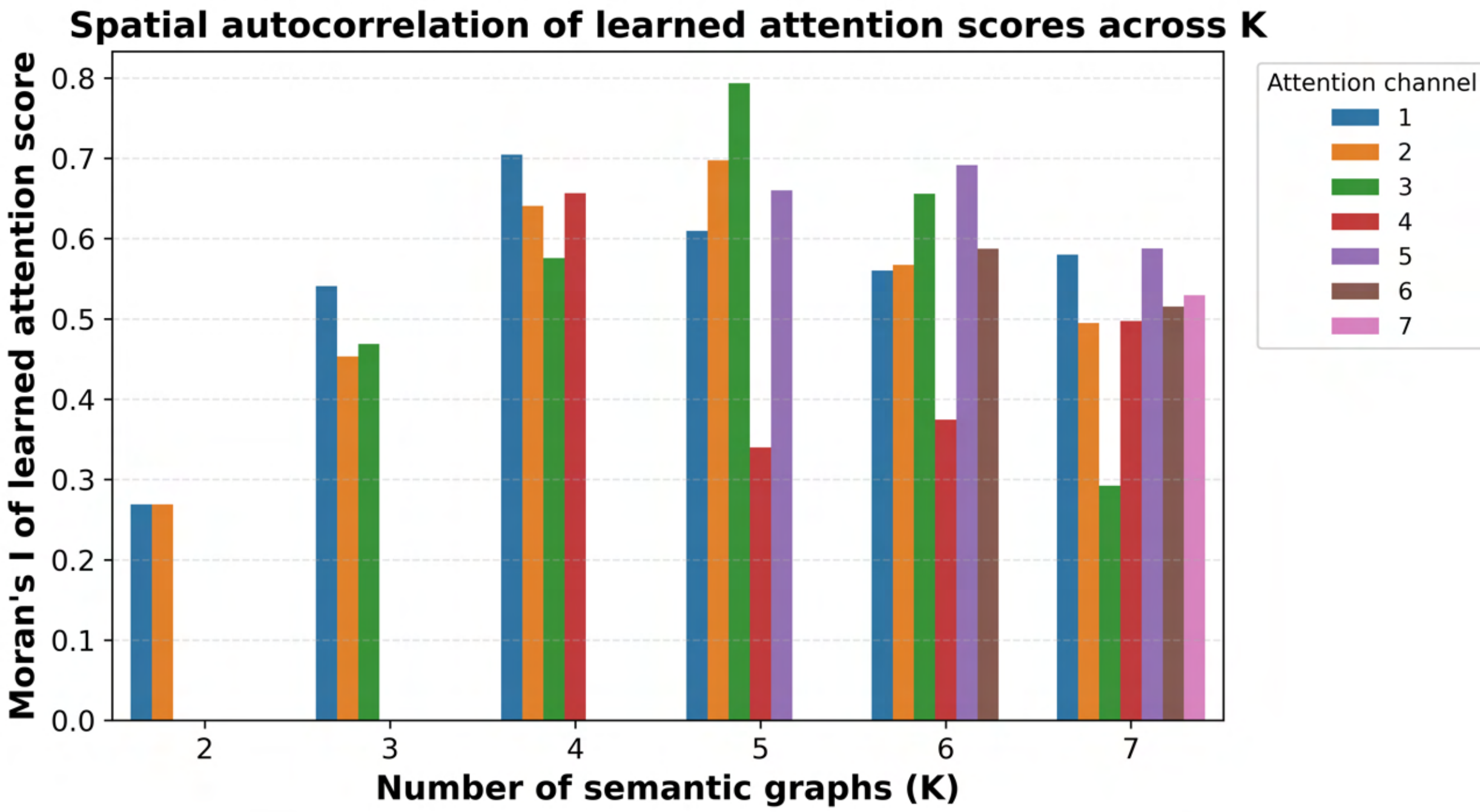

Figure S25: Spatial autocorrelation of learned attention scores for each semantic graph channel across $K = 2$ to $K = 7$ on the Mouse Olfactory Bulb dataset (Stereo-seq). Each bar represents the Moran's I score of one attention channel (semantic graph) for a given K. At $K = 4$, three channels achieve consistently high Moran's I values (0.58–0.70). At $K = 5$ and beyond, the scores become increasingly inconsistent across channels — one channel reaches a high value while others decline — indicating that additional semantic graphs capture less spatially structured biological signals. This pattern supports $K = 4$ as the point at which the model extracts the maximum number of spatially coherent semantic views.

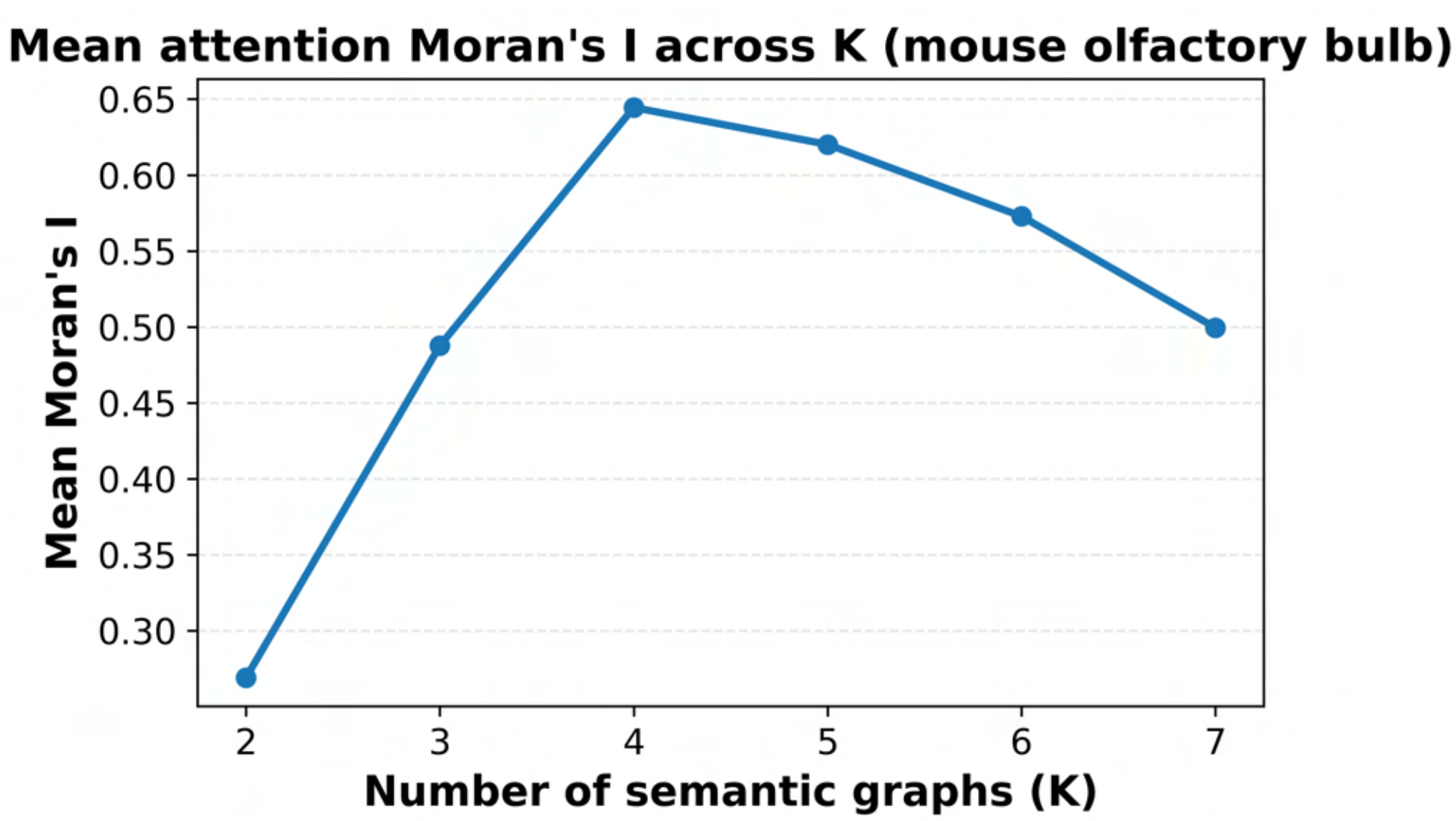

Figure S26: Mean Moran's I of learned attention scores across $K = 2$ to $K = 7$ on the Mouse Olfactory Bulb dataset (Stereo-seq). The peak at $K = 4$ identifies the saturation point beyond which additional semantic graphs no longer capture spatially coherent biological signals, consistent with the $\mathcal{O}(K^2)$ computational scaling of the disentanglement loss.

**Method / Regime**	**ARI**	**NMI**	**1-Hop Coverage (%)**
Expression-only baseline (PCA, 50 PCs)	0.22	0.35	–
Novae	0.46	0.64	–
SemanticST, full-batch	0.35	0.63	99.4
SemanticST, mini-batch (batch 10,000)	0.39	0.63	10.37
SemanticST, mini-batch (batch 5,000)	0.47	0.65	5.38
SemanticST, mini-batch (batch 3,000)	**0.51**	**0.65**	3.26

Table S1: Clustering performance on the STARmap dataset (N = 91,246 cells, 16 annotated domains), scored against the expert annotation. All SemanticST runs use the encoder output with mclust fixed to 16 domains. 1-hop coverage is the fraction of each cell's true full-tissue neighbours retained within its reconstructed batch graph.

**Method / Regime**	**ARI**	**NMI**	**1-Hop Coverage (%)**
Expression-only (PCA)	0.0006	0.0012	–
Novae	0.89	0.89	–
SemanticST, mini-batch (3,000)	0.977	0.962	1.06
SemanticST, mini-batch (10,000)	0.967	0.972	7.00
SemanticST, mini-batch (20,000)	**0.983**	**0.972**	17.04

Table S2: Clustering performance on the large synthetic benchmark (N = 282,694 cells, 200 genes) against ground-truth domain labels.

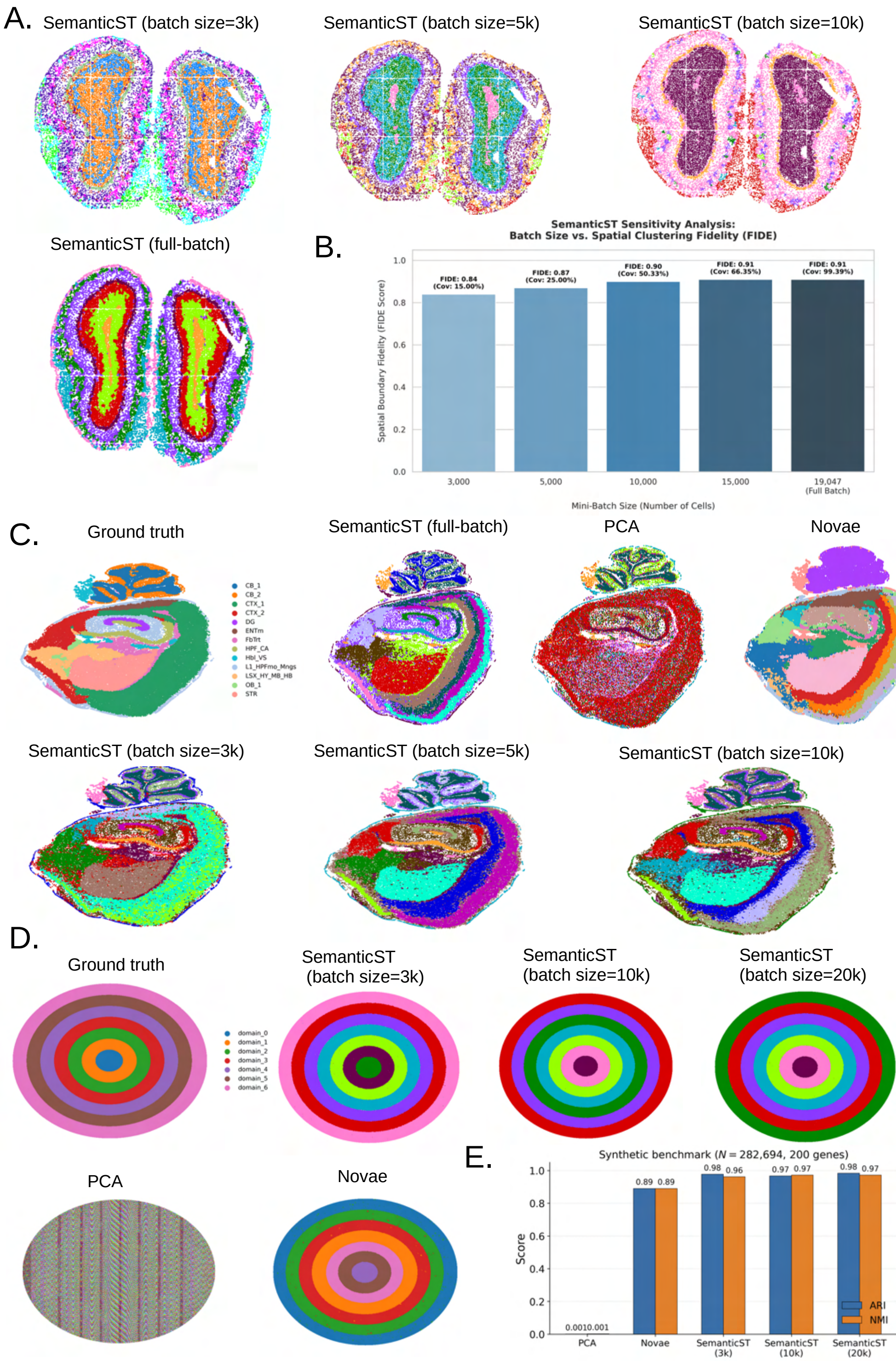

Figure S27: Mini-batch training preserves spatial domains across scales. **(A)** Spatial domains recovered by SemanticST on the Mouse Olfactory Bulb dataset (N = 19,047) under mini-batch training (batch sizes 3k, 5k, 10k) and full-batch training. **(B)** Spatial boundary fidelity (FIDE) and 1-hop true-neighbour coverage as a function of batch size on the same dataset. **(C)** STARmap mouse tissue (N = 91,246); ground truth, SemanticST (full-batch and batch sizes 3k, 5k, 10k), PCA, and Novae. **(D)** Large synthetic benchmark (N = 282,694); ground truth, SemanticST (batch sizes 3k, 10k, 20k), PCA, and Novae. **(E)** ARI and NMI on the synthetic benchmark.

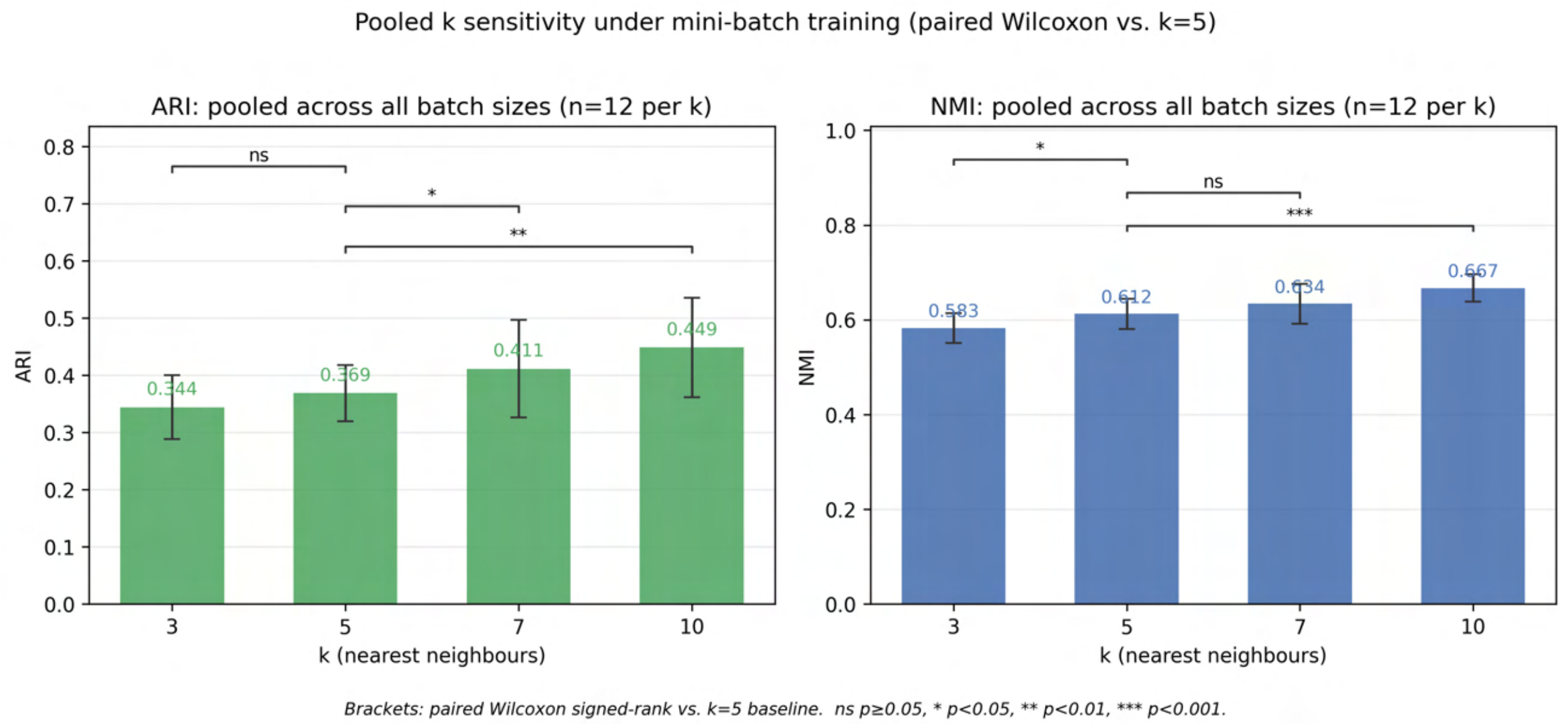

Figure S28: Sensitivity to k under mini-batch training on STARmap PLUS, pooled across batch sizes ($n = 12$ per k). Bars: mean ARI (left) and NMI (right) ±SD; brackets: paired Wilcoxon vs. $k = 5$ (ns $p \geq 0.05$, $*\ p < 0.05$, $**$ $p < 0.01$, $***\ p < 0.001$).

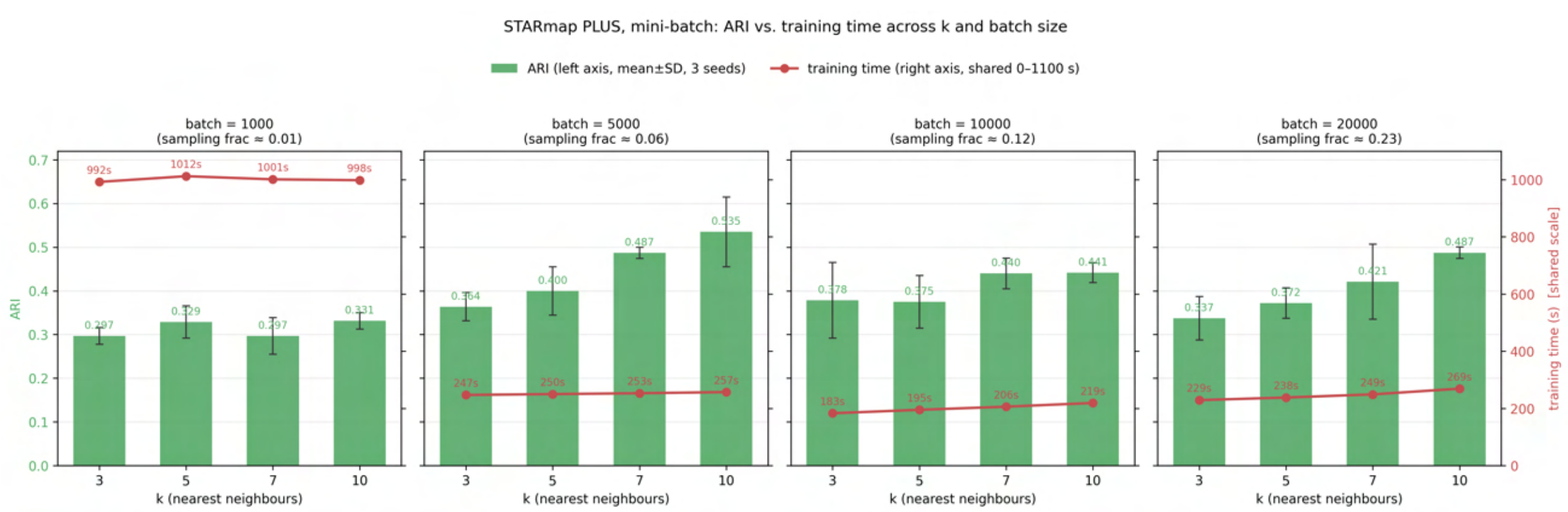

Figure S29: Clustering quality and training cost under mini-batch training (STARmap PLUS). One panel per batch size; bars: mean ARI (±SD, 3 seeds) vs. k; red line: mean training time (shared 0–1100 s scale).

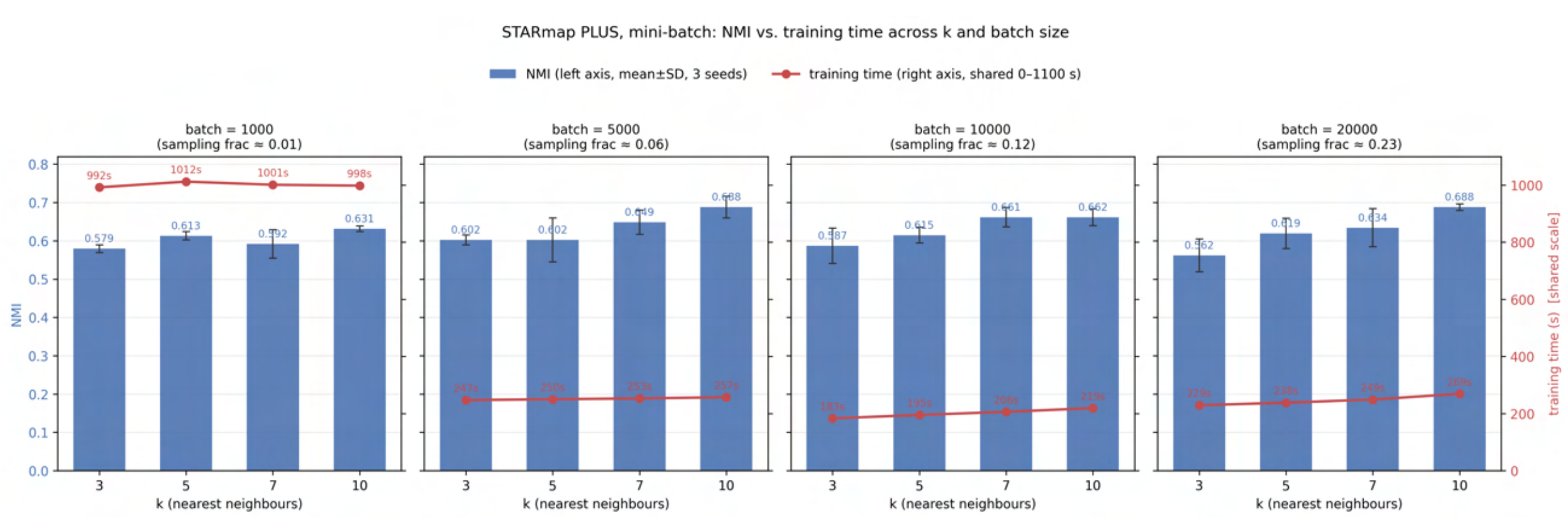

Figure S30: Clustering quality and training cost under mini-batch training (STARmap PLUS). One panel per batch size; bars: mean NMI (±SD, 3 seeds) vs. k; red line: mean training time (shared 0–1100 s scale).

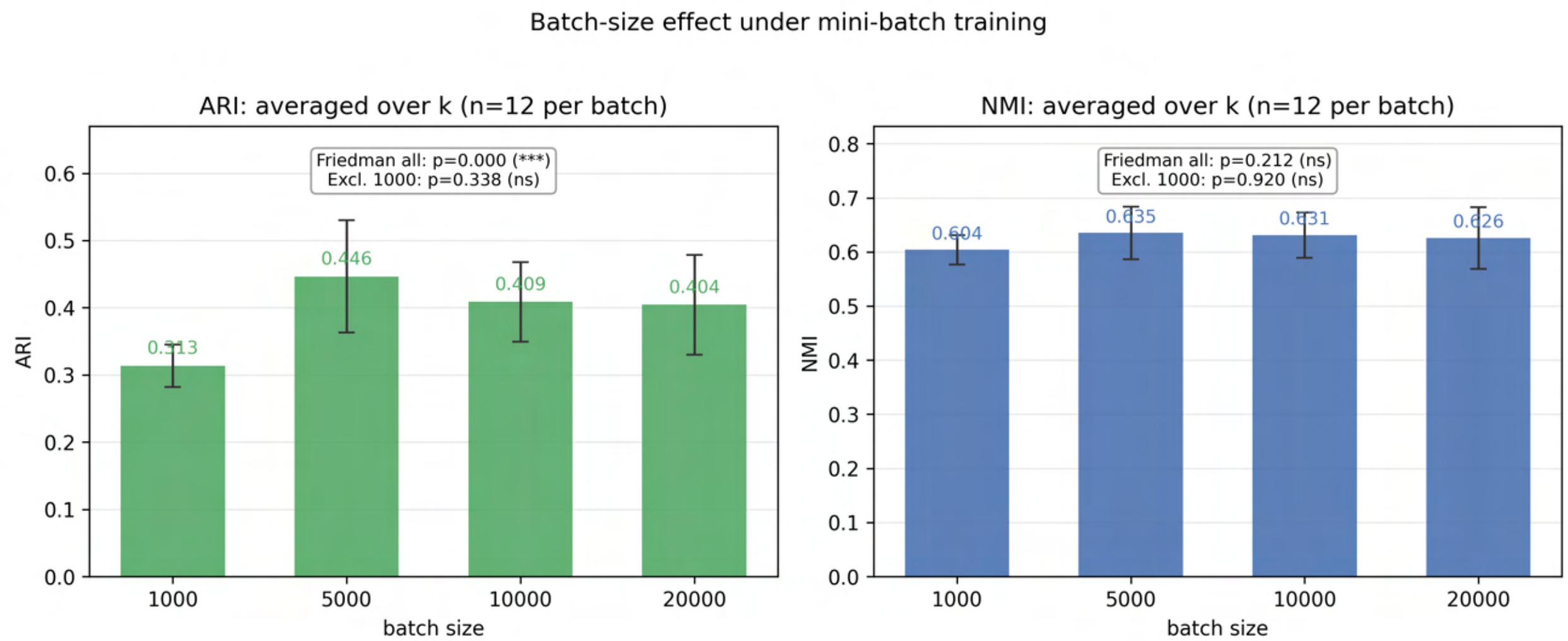

Figure S31: Effect of mini-batch size on clustering quality (STARmap PLUS), averaged over k ($n = 12$ per batch size: 4 values of $k \times 3$ seeds). Bars show mean ARI (left) and NMI (right) ±SD. Inset: Friedman tests across batch sizes (blocked by k and seed); a significant effect appears only when the smallest batch ($B = 1000$) is included, and disappears once it is excluded ($B \in \{5000, 10000, 20000\}$: ARI $p = 0.34$, NMI $p = 0.92$)

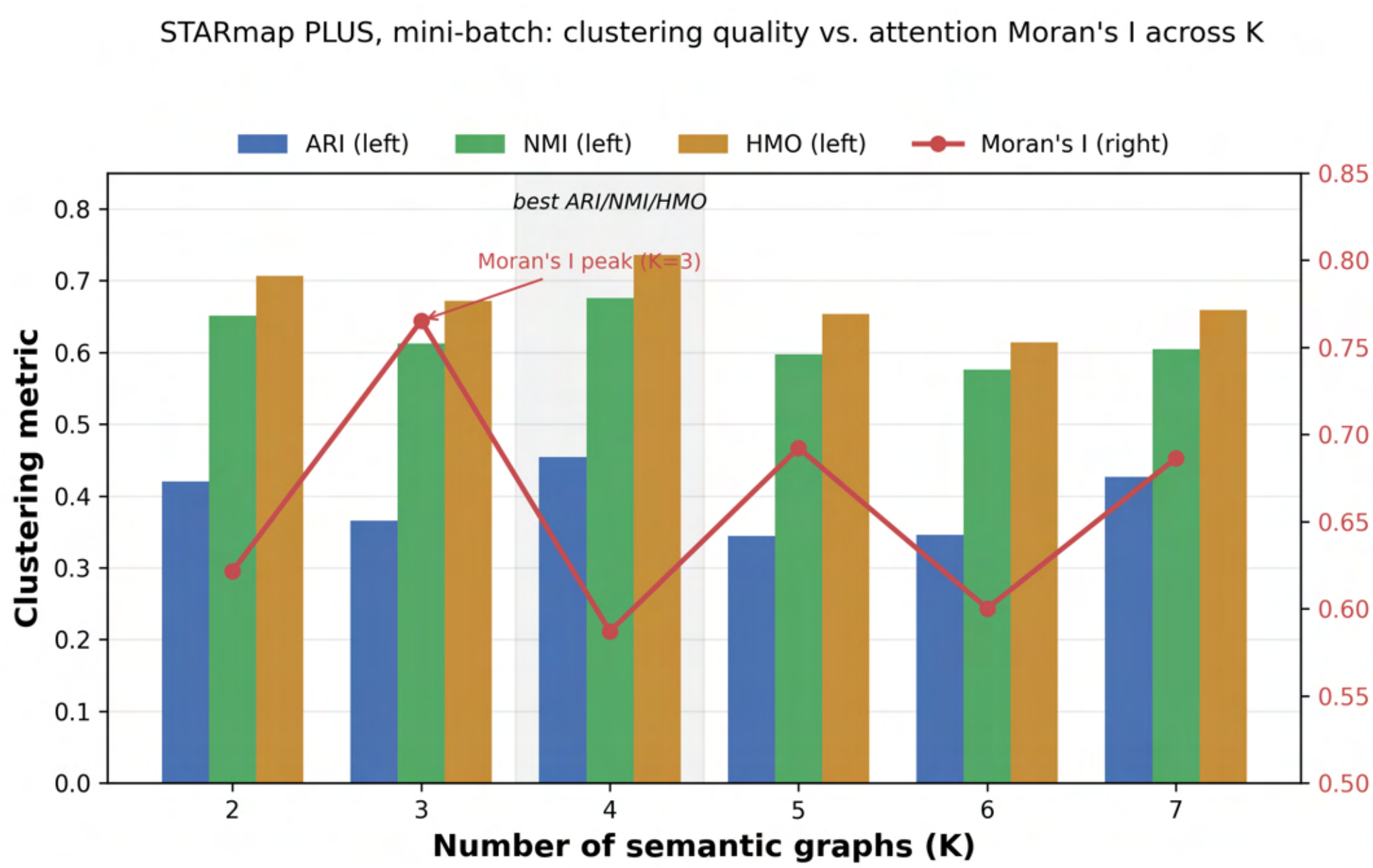

Figure S32: Sensitivity to the number of semantic graphs K under mini-batch training on STARmap PLUS. Bars show downstream clustering quality against the 16 annotated domains (ARI, NMI, HMO; left axis) and the red line shows the mean attention-level Moran's I computed on the full-tissue spatial graph (right axis).

Platform	Tissue	Section	#Spot/cell	Reference
**10X Visium**	**Human dorsolateral prefrontal cortex**	151507	4,226	[1]
		151508	4,384	
		151509	4,789	
		151510	4,634	
		151669	3,661	
		151670	3,498	
		151671	4,110	
		151672	4,015	
		151673	3,639	
		151674	3,573	
		151675	3,592	
		151676	3,460	
	**Mouse brain**	Mouse Brain Section 1 (Sagittal-Anterior)	2,695	10x Visium demo
		Mouse Brain Section 1 (Sagittal-Posterior)	3,355	
	**Human breast**	Human Breast Cancer Section 1	3,798	
	**Mouse brain**	Coronal section	2,902	
	**Mouse breast**	S1_A1 Untreated breast tumor slice #1 from mouse NC3	1,868	[2]
		S3_A1 Untreated breast tumor slice #2 from mouse	1,950	
Xenium	**Human Breast**	Human breast cancer	167,780	[3]
Stereo-seq	**Mouse embryo**	E9.5	5,913	[4]
	**Mouse olfactory bulb**	Puck_200127_15	19,109	
Slide-seqV2	**Mouse hippocampus**	Puck_200115_08	52,869	[5]

Table S3: A detailed overview of all the spatial transcriptomics datasets utilized in this study.

References

[1] Kristen R Maynard, Leonardo Collado-Torres, Lukas M Weber, Cedric Uytingco, Brianna K Barry, Stephen R Williams, Joseph L Catallini, Matthew N Tran, Zachary Besich, Madhavi Tippani, et al. Transcriptome-scale spatial gene expression in the human dorsolateral prefrontal cortex. *Nature neuroscience*, 24(3):425–436, 2021.

[2] Yahui Long, Kok Siong Ang, Mengwei Li, Kian Long Kelvin Chong, Raman Sethi, Chengwei Zhong, Hang Xu, Zhiwei Ong, Karishma Sachaphibulkij, Ao Chen, et al. Spatially informed clustering, integration, and deconvolution of spatial transcriptomics with graphst. *Nature Communications*, 14(1):1155, 2023.

[3] Amanda Janesick, Robert Shelansky, Andrew D Gottscho, Florian Wagner, Stephen R Williams, Morgane Rouault, Ghezal Beliakoff, Carolyn A Morrison, Michelli F Oliveira, Jordan T Sicherman, et al. High resolution mapping of the tumor microenvironment using integrated single-cell, spatial and in situ analysis. *Nature communications*, 14(1):8353, 2023.

[4] Ao Chen, Sha Liao, Mengnan Cheng, Kailong Ma, Liang Wu, Yiwei Lai, Xiaojie Qiu, Jin Yang, Jiangshan Xu, Shijie Hao, et al. Spatiotemporal transcriptomic atlas of mouse organogenesis using dna nanoball-patterned arrays. *Cell*, 185(10):1777–1792, 2022.

[5] Author Name or Broad Institute. Slide-seq study, 2023. Accessed: 2023-02-25.
